# The prelimbic cortex regulates itch processing by controlling attentional bias

**DOI:** 10.1016/j.isci.2022.105829

**Published:** 2022-12-17

**Authors:** Guang-Yan Wu, Xiao-Xia Zheng, Shan-Lan Zhao, Yi Wang, Shan Jiang, Yi-Song Wang, Yi-Lun Yi, Juan Yao, Hui-Zhong Wen, Ju Liu, Hong-Li Li, Jian-Feng Sui

**Affiliations:** 1 Experimental Center of Basic Medicine, College of Basic Medical Sciences, Army Medical University, Chongqing 400038, China; 2 Department of Physiology, College of Basic Medical Sciences, Army Medical University, Chongqing 400038, China; 3 Department of Neurobiology, College of Basic Medical Sciences, Chongqing Key Laboratory of Neurobiology, Army Medical University, Chongqing 400038, China; 4 Department of Foreign Languages, College of Basic Medical Sciences, Army Medical University, Chongqing 400038, China

**Keywords:** Biological sciences, Neuroscience, Cell biology

## Abstract

Itch is a complex and unpleasant sensory experience. Recent studies have begun to investigate the neural mechanisms underlying the modulation of sensory and emotional components of itch in the brain. However, the key brain regions and neural mechanism involved in modulating the attentional processing of itch remain elusive. Here, we showed that the prelimbic cortex (PrL) is associated with itch processing and that the manipulation of itch-responsive neurons in the PrL significantly disrupted itch-induced scratching. Interestingly, we found that increasing attentional bias toward a distracting stimulus could disturb itch processing. We also demonstrated the existence of a population of attention-related neurons in the PrL that drive attentional bias to regulate itch processing. Importantly, itch-responsive neurons and attention-related neurons significantly overlapped in the PrL and were mutually interchangeable in the regulation of itch processing at the cellular activity level. Our results revealed that the PrL regulates itch processing by controlling attentional bias.

## Highlights

• PrL is involved in itch processing
• A distracting stimulus can disturb itch processing
• Itch-responsive and attention-related neurons significantly overlap in the PrL
• The two neuronal populations are interchangeable at the cellular activity level

## Graphical abstract

**Figure:**
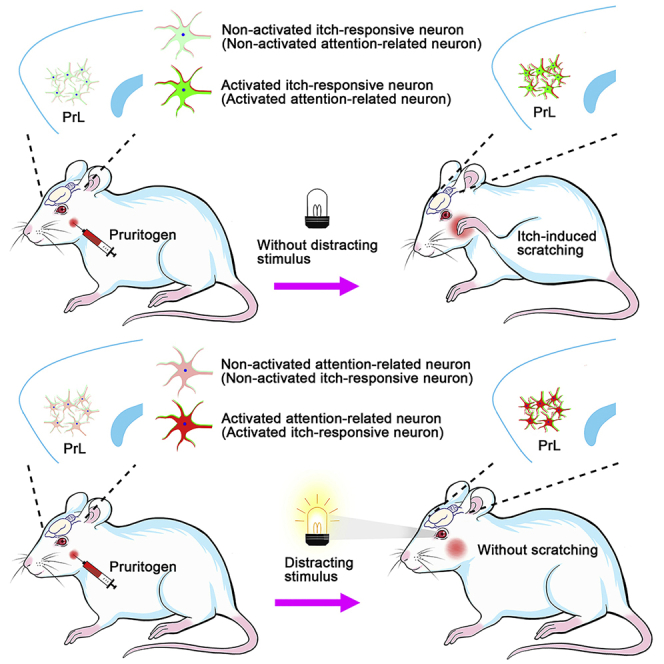

## Introduction

Itch, or pruritus, is defined as the unpleasant sensation that evokes a desire to scratch and serves as a physiological mechanism for self-protection against harmful external threats.^1^^,^^2^ However, under pathological conditions, such as in skin diseases such as atopic dermatitis and other systemic disorders, including liver and kidney diseases, itch becomes more severe and chronic, which leads to excessive, repetitive scratching.^3^^,^^4^^,^^5^ Such long-term out-of-control scratching can lead to serious skin and tissue damage, which can worsen the itch sensation and lead to further scratching.^6^^,^^7^^,^^8^^,^^9^ Unfortunately, current treatments for itch are largely ineffective. Thus, a deeper understanding of the mechanism underlying itch is urgently needed.

Itch is a complex sensory and emotional experience regulated by neural networks that encode sensory, emotional, attentional, evaluation, and motivational information.^10^^,^^11^ The spinothalamic tract and spinoparabrachial pathway are two major pathways involved in itch sensation transmission.^6^^,^^12^ Moreover, recent studies have further explored the possible neural mechanisms underlying the emotional component of itch sensation. For example, a recent study showed that periaqueductal gray (PAG) GABAergic but not glutamatergic neurons encode the aversive component of itch.^13^ A previous study also found that itch-responsive amygdala neurons play a critical role in encoding the negative emotional component of itch sensation.^14^ Additionally, ventral tegmental area (VTA) GABAergic neurons play a critical role in encoding the aversive component of itch sensation.^9^ Importantly, unlike that of VTA GABAergic neurons, itch-induced activation of VTA dopaminergic (DA) neurons lags behind the onset of a scratching train by several seconds, and VTA DA neurons contribute to the pleasure associated with scratching an itch.^9^ These studies suggest that multiple brain regions are involved in regulating the emotional components of itch sensation.

Attention serves as a gatekeeper, allowing the processing and prioritization of signals according to their relevance or saliency.^15^^,^^16^ Because of the aversive and interruptive characteristics of itch, attention appears to play a crucial role in itch processing.^16^^,^^18^^,^^19^^,^^17^ Indeed, a number of studies have reported that attentional processing is related to itch, further confirming that attentional bias is related to itch in humans.^16^^,^^19^ Human neuroimaging studies have also revealed significant changes in the activity of the dorsolateral prefrontal cortex associated with attention-dependent processing in healthy volunteers with histamine-induced itch and atopic dermatitis patients.^11^^,^^20^ However, to date, there is little information about the cellular and neural circuit mechanisms underlying the key role of the attention component in itch processing.

Neuroimaging studies have revealed that the prefrontal cortex, which is associated with cognition and attention, is activated during the itch-scratching cycle.^11^^,^^20^^,^^21^ The prelimbic cortex (PrL) of the medial prefrontal cortex plays an important role in attention regulation in rats.^22^^,^^23^^,^^24^ For instance, the PrL has been shown to be involved in modulating attention in fear learning and spatial memory.^22^^,^^23^ Thus, in this study, we investigated the potential role of the PrL in regulating the attentional processing of itch.

## Results

### The prelimbic cortex was involved in itch processing

**Figure 1:**
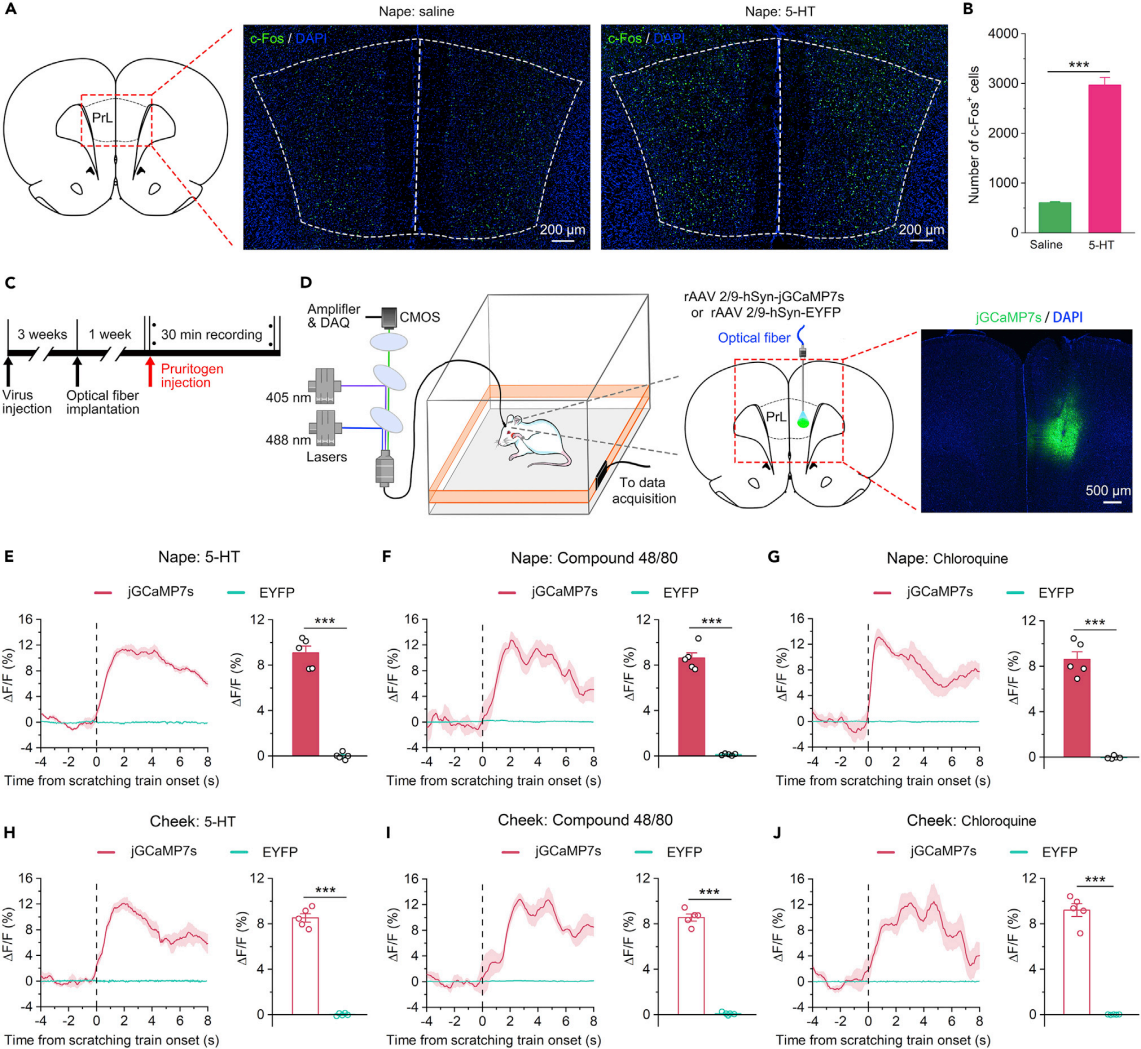
Neural activity in the PrL was significantly enhanced during itch-induced scratching (A) Representative immunofluorescence staining image of c-Fos^+^ neurons in the PrL after intradermal injection of 5-HT or saline into the nape of the neck. (B) Summary of the data showing the number of c-Fos + neurons in subareas of the PrL. Saline: n = 3 rats; 5-HT: n = 4 rats. (C) Schematic diagram showing the basic experimental timeline of fiber photometry experiments. (D) Left: Schematic illustration of the recording system used to obtain calcium fluorescence signals. Right: Schematic showing the injection of rAAV 2/9-hSyn-jGCaMP7s or rAAV 2/9-hSyn-EYFP and implantation of an optical fiber into the right PrL of the rats. Representative images of jGCaMP7s expression. (E–J) Left: Peri-event plots of the average calcium transients of global PrL neurons in response to itch-scratching induced by intradermal injection of 5-HT (nape, E; cheek, H), compound 48/80 (nape, F; cheek, I), or chloroquine (nape, G; cheek, J). The thick lines and shaded regions indicate the mean ± SEM. Right: Quantification of average jGCaMP7s and EYFP fluorescence changes during itch-induced scratching behaviors (from 0 s to 6 s). n = 5 rats in each group. The data are presented as the mean ± SEM and have a normal distribution. Two-tailed Unpaired Student’s *t* test; ∗∗∗p < 0.001.

First, to explore the functional role of the PrL in itch processing, we examined the activation of PrL neurons by using c-Fos as a neuronal activity marker. We found that the number of c-Fos^+^ neurons increased distinctly in rats that received intradermal injection of pruritogen 5-HT into the nape of the neck compared to saline-treated rats (Figures 1A and 1B), suggesting that itch evoked by 5-HT activated neurons in the PrL. In addition, by inducing the expression of the genetically encoded calcium indicator jGCaMP7s in the PrL, we were able to record intracellular Ca^2+^ transients in these neurons in behavioral rats to investigate the temporal dynamics of these PrL neurons.^25^^,^^26^ rAAV 2/9-hSyn-jGCaMP7s (or rAAV-2/9-hSyn-EYFP as a control) was injected unilaterally into the PrL (Figure 1C), and the indicator jGCaMP7s or EYFP was expressed in the PrL (Figure 1D). An optical fiber was implanted unilaterally into the PrL to measure changes in Ca^2+^ dynamics after intradermal injection of different pruritogens (5-HT, compound 48/80, or chloroquine) into the nape of the neck or cheek during behavioral testing. As expected, we found that PrL neurons showed significant and long-lasting increases in Ca^2+^ transients during pruritogen-evoked scratching beginning at the onset of scratching behaviors (Figures 1E–1J). These results suggest that PrL is strongly associated with itch processing.

**Figure 2:**
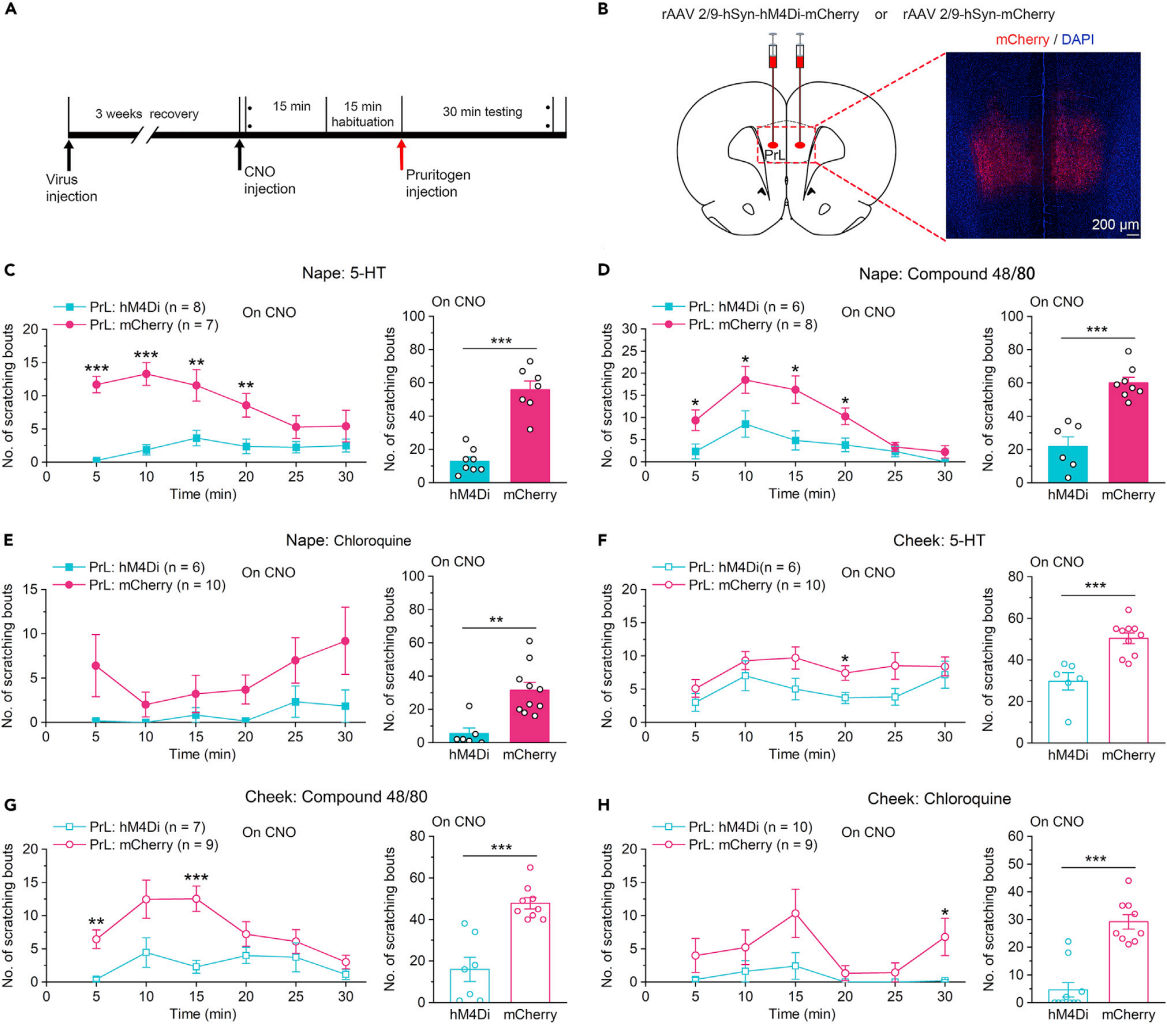
Pharmacogenetic inhibition of the PrLsuppressed itch processing (A) Flow diagram of the basic experimental timeline for chemogenetic inhibition. (B) Schematic showing bilateral injection of rAAV 2/9-hSyn-hM4Di-mCherry or rAAV 2/9-hSyn-mCherry into the PrL of rats (left). Representative images of mCherry expression (right). (C–E) Pharmacogenetic inhibition of the PrL significantly impaired scratching induced by intradermal injection of 5-HT (C), compound 48/80 (D), or chloroquine (E) into the nape of the neck. n = 6–10 rats in each group. (F–H) Pharmacogenetic inhibition of the PrL significantly disrupted scratching induced by intradermal injection of 5-HT (F), compound 48/80 (G), or chloroquine (H) into the cheek. n = 6–10 rats in each group. The data are presented as the mean ± SEM and have a normal distribution. Two-way repeated measures ANOVA followed by the separate one-way ANOVA, or two-tailed Unpaired Student’s *t* test; ∗p < 0.05, ∗∗p < 0. 01, ∗∗∗p < 0.001.

Next, the function of PrL was further validated by pharmacogenetic methods.^27^ We used clozapine-oxide (CNO) to reliably modulate the activity of neurons expressing designer receptors exclusively activated by designer drugs (DREADDs). rAAV 2/9-hSyn-hM4Di-mCherry (or rAAV 2/9-hSyn-mCherry as a control) was bilaterally injected into the PrL (Figure 2B). The efficacy of hM4Di-mediated inhibition has been confirmed in our recent study.^28^ Then, we tested the scratching behaviors evoked by intradermal injection of pruritogens (5-HT, compound 48/80, or chloroquine) into the nape of the neck 30 min after intraperitoneal (i.p.) injection of CNO (Figure 2B). Our results showed that the pharmacogenetic inhibition of PrL neurons markedly decreased the number of scratching bouts induced by 5-HT, compound 48/80, or chloroquine (Figures 2C–2E). Moreover, in the cheek model of itch, the pharmacogenetic inhibition of PrL neurons also significantly decreased the number of scratching bouts induced by 5-HT or compound 48/80, chloroquine (Figures 2F–2H). These results suggest that PrL neurons play an important role in itch processing.

**Figure 3:**
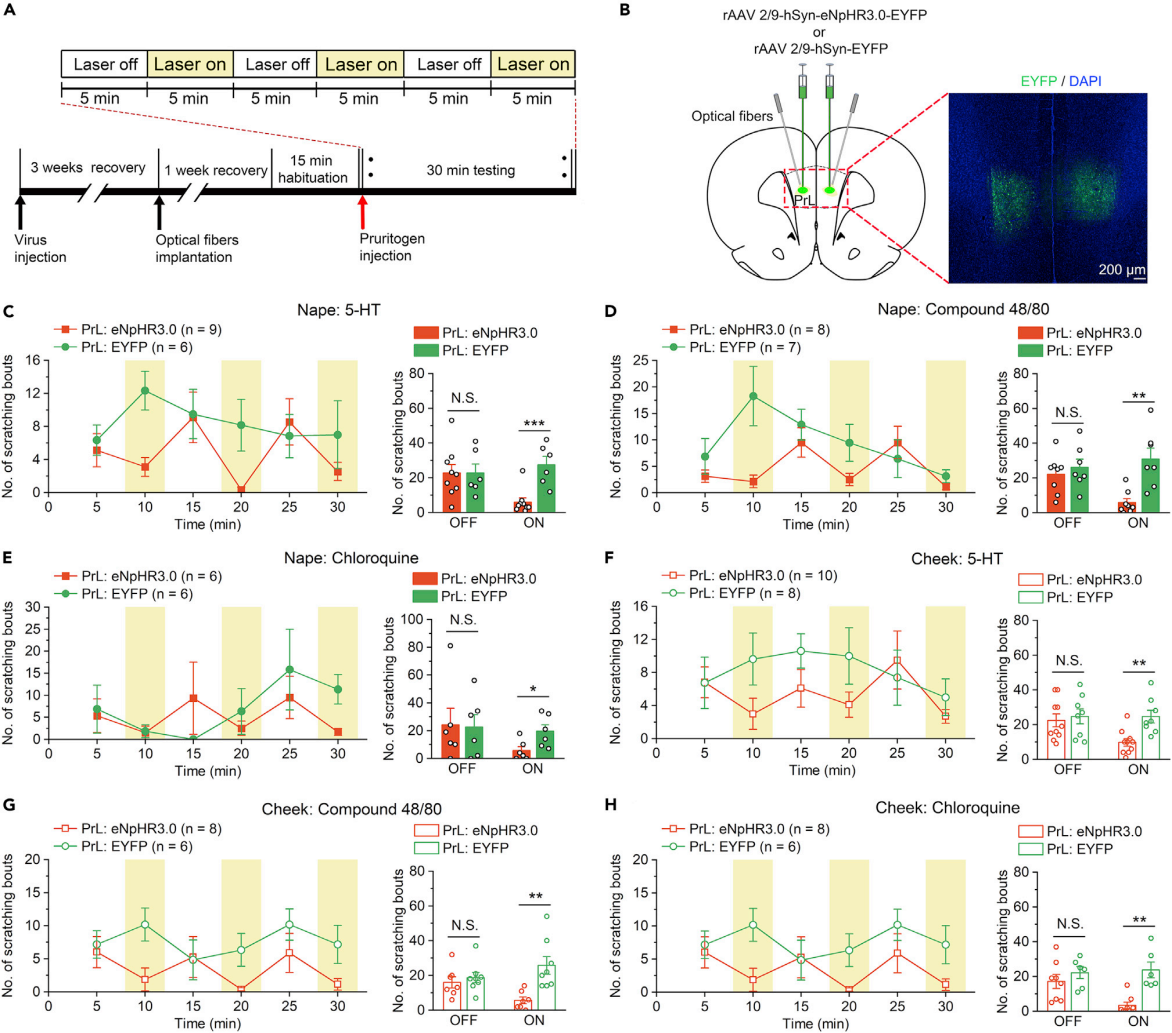
Optogenetic inhibition of the PrL impaired itch processing (A) Flow diagram of the basic experimental timeline for optogenetic inhibition. (B) Schematic showing injection of rAAV 2/9-hSyn-eNpHR3.0-EYFP or rAAV 2/9-hSyn-EYFP and optical fibers implantation in the bilateral PrL of rats (left). Representative images of EYFP expression (right). (C–E) Optogenetic inhibition of the PrL significantly decreased the scratching bouts induced by intradermal injection of 5-HT (C), compound 48/80 (D), or chloroquine (E) into the nape of the neck. n = 6–9 rats in each group. (F–H) Optogenetic inhibition of the PrL also significantly impaired the scratching induced by intradermal injection of 5-HT (F), compound 48/80 (G), or chloroquine (H) into the cheek. n = 6–10 rats in each group. The data are presented as the mean ± SEM and have a normal distribution. Two-tailed Unpaired Student’s *t* test; N.S., not significant, ∗p < 0.05, ∗∗p < 0. 01, ∗∗∗p < 0.001.

In addition, optogenetics is one of the most effective approaches for investigating the neural mechanisms underlying rodent behaviors with high temporal and spatial precision.^29^^,^^30^^,^^31^ We further confirmed the effects of PrL neurons inhibition on itch by using optogenetic manipulation. rAAV 2/9-hSyn-eNpHR3.0-EYFP (or rAAV 2/9-hSyn-EYFP as a control) was bilaterally injected into the PrL, and two optic fibers were bilaterally implanted into the PrL at a 15-degree angle three weeks later (Figure 3B). The efficacy of eNpHR3.0-mediated inhibition has been confirmed in our recent study.^28^ In the behavioral tests, to effectively inhibit PrL neurons and avoid damage to brain tissue caused by long-lasting illumination, we used a stimulation cycle involving 5 min of illumination (593 nm) and 5 min intervals of rest. Pruritogens (5-HT, or compound 48/80, chloroquine) were intradermally injected into the cheek and nape of the neck 5 min before receiving delivery of laser (593 nm) to the PrL, and scratching behaviors were recorded for 30 min with cycles of laser off and laser on (Figure 3A). Consistently, we found that the optogenetic inhibition of the PrL neurons also significantly reduced the number of scratching bouts induced by 5-HT, compound 48/80, or chloroquine during the laser-on periods (Figures 3C–3H). However, there were no significant differences in the number of scratching bouts between the eNpHR3.0-expressing and EYFP-expressing rats during the laser-off periods (Figures 3C–3H). Taken together, these results suggest that the PrL plays a critical role in the modulation of itch-induced scratching behaviors.

### Prelimbic cortex itch-responsive neurons were involved in itch-induced scratching behaviors

Based on the above results, the PrL is involved in the cerebral processing of itch. However, the specific role of the PrL in modulating itch processing is still unclear. To further explore the role of the PrL in regulating itch processing, we labeled itch-responsive neurons in the PrL that were activated and may specifically contribute to itch processing. The promoter of c-Fos, an immediate-early gene often used as a marker of recent neuronal activity, was coupled to the tetracycline transactivator (tTA), a key component of the doxycycline (Dox) system for inducible expression of a gene of interest.^32^^,^^33^^,^^34^^,^^35^^,^^36^ First, we carried out several sets of experiments to test the reliability of the Tet-Off system. We bilaterally coinjected rAAV 2/9-*c*-Fos-tTA and rAAV 2/9-TRE-tight-mCherry virus into the PrL (Figure S2C). In this system, the presence of Dox inhibited c-Fos promoter-driven binding of tTA to its target tetracycline-responsive element (TRE) site, which in turn prevented it from driving mCherry expression (Figures S2A and S2B). Histological experiments showed that 82.67% of PrL neurons expressed mCherry in rats without a Dox diet, whereas continuous Dox diet feeding stably blocked the activation of the TRE promoter (Figures S2D and S2E). In addition, after 3 days of stopping the Dox diet, mCherry expression induced by 5-HT was significantly higher than that induced by normal saline (Figures S2D and S2E), suggesting that the Tet-Off system can be used to label specific neurons during itch processing. Furthermore, there was no significant difference in mCherry expression at 3 days after pruritogen labeling and 14 days after pruritogen labeling (Figures S2D and S2E), indicating that virus-expressed mCherry could persist for at least 14 days and did not change significantly in rats used in behavioral experiments performed at later time points.

**Figure 4:**
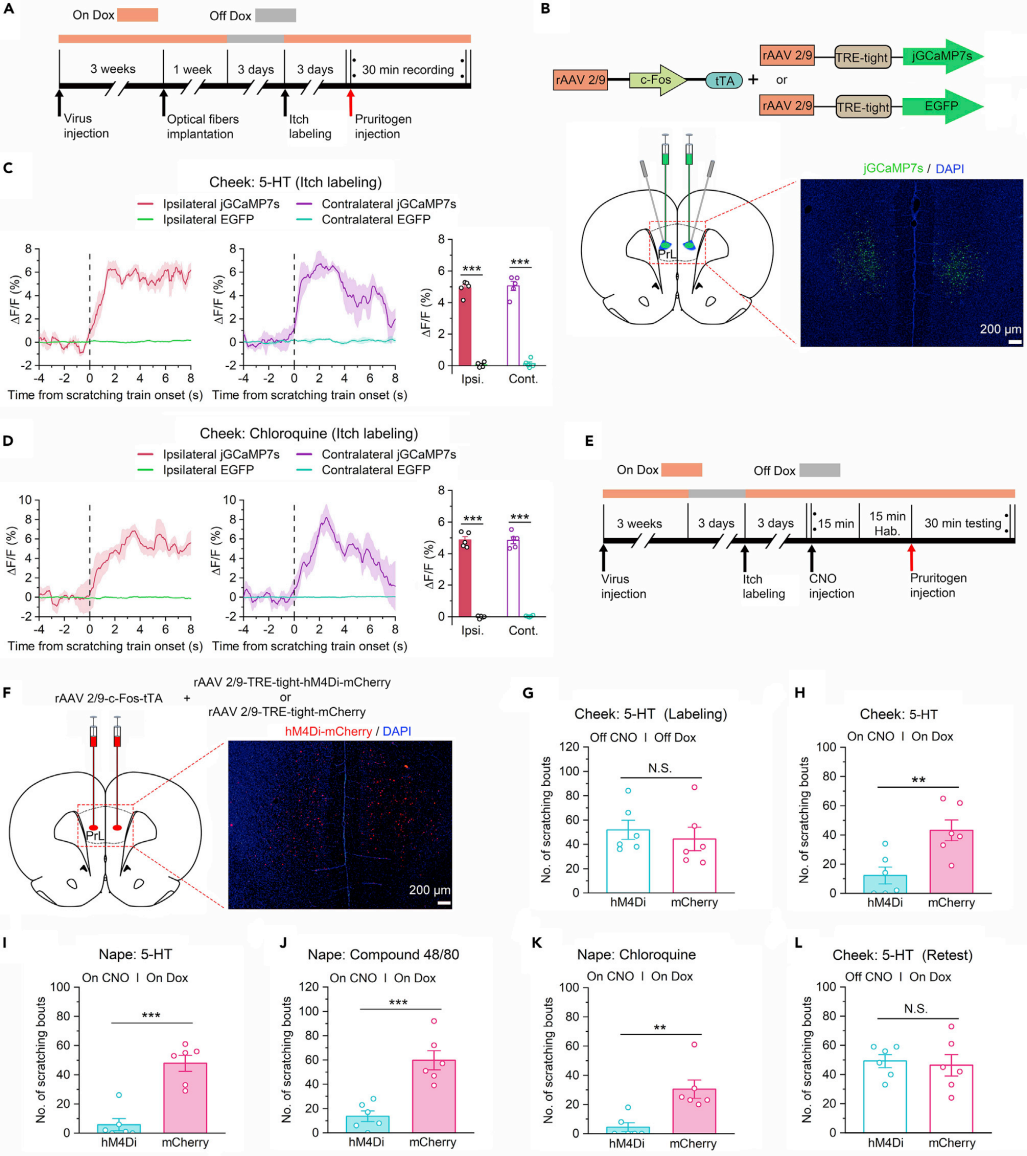
Pharmacogenetic inhibition of itch-responsive neurons in the PrL impaired itch processing (A) Basic schematic of virus-based activity-dependent labeling and fiber photometry experiments. (B) Upper: Schematic showing the mixture of rAAV 2/9-*c*-Fos-tTA and rAAV 2/9-TRE-tight-jGCaMP7s (or rAAV 2/9-TRE-tight-EGFP). Lower: Rats were injected with virus mixtures, and optical fibers were bilaterally implanted in the PrL. Representative images of jGCaMP7s expression. (C and D) Left and middle: Mean fluorescent signal of PrL itch-responsive neurons in response to itch-scratching induced by intradermal injection of 5-HT (C) or chloroquine (D). The thick lines and shaded regions indicate the mean ± SEM. Right: Quantification of average jGCaMP7s and EGFP fluorescence changes during itch-induced scratching behaviors (from 0 s to 6 s). n = 5 rats in each group. (E) Basic schematic of virus-based activity-dependent labeling and pharmacogenetic inhibition experiments. (F) Left: Schematic showing bilateral injection of rAAV 2/9-*c*-Fos-tTA and rAAV 2/9-TRE-tight-hM4Di-mCherry (or rAAV2/9-TRE-tight- mCherry) into the PrL of rats. Right: Representative images of hM4Di-mCherry expression. (G) After 3 days without Dox diet feeding, rats were injected with 5-HT into the cheek to label itch-responsive neurons in the PrL. n = 6 rats in each group. (H–K) Chemogenetic inhibition of itch-responsive neurons significantly decreased the number of scratching bouts induced by intradermal injection of 5-HT (H, cheek; I, nape), compound 48/80 (J, nape), or chloroquine (K, nape). n = 6 rats in each group. (L) Rats were fed with a Dox diet and injected with 5-HT in the absence of CNO to reassess the decrease in the number of scratching bouts resulting from chemogenetic inhibition. n = 6 rats in each group. The data are presented as the mean ± SEM and have a normal distribution. Two-tailed Unpaired Student’s *t* test; N.S., not significant, ∗∗p < 0. 01, ∗∗∗p < 0.001.

**Figure 5:**
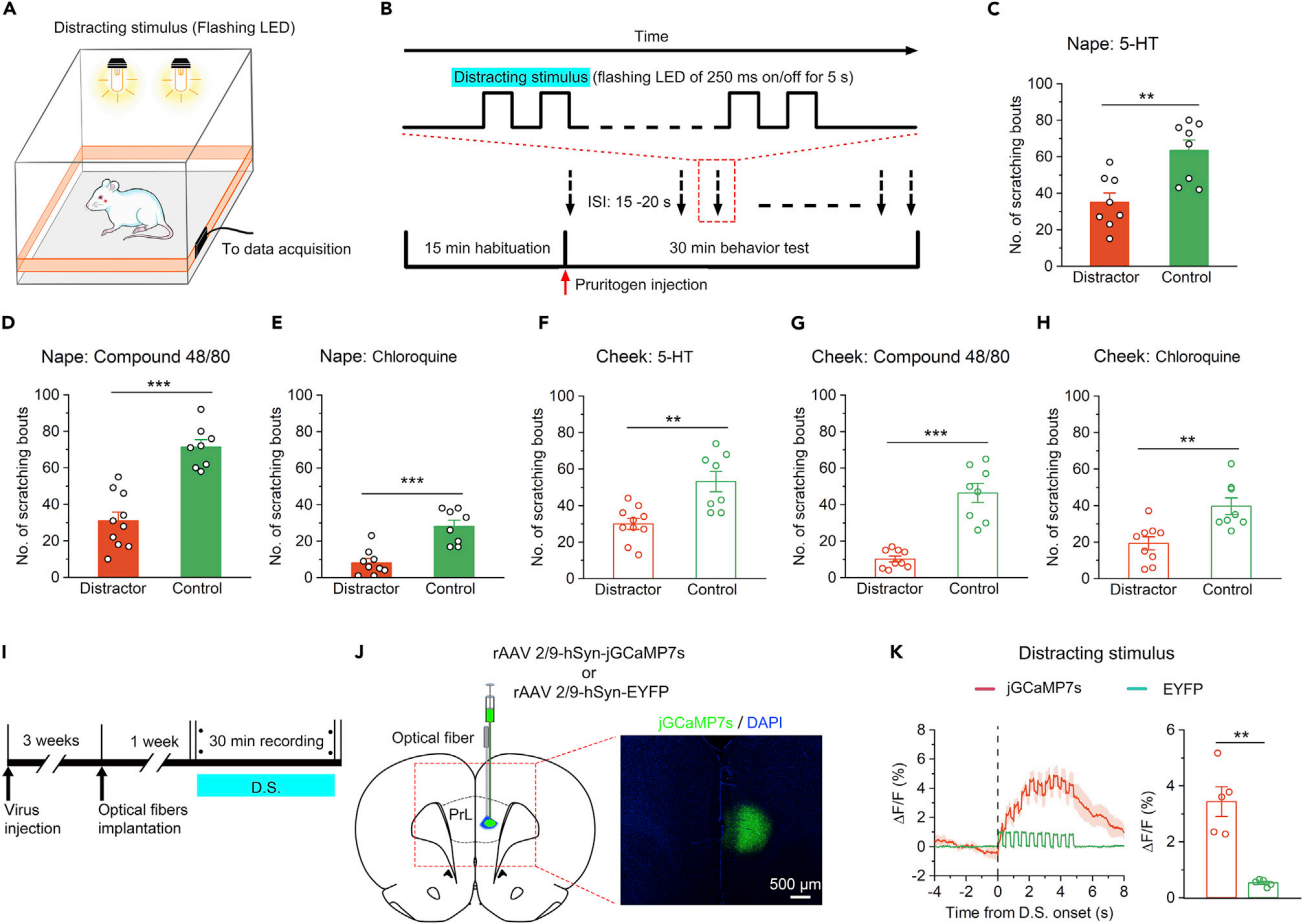
The distracting stimulus significantly disrupted itch processing and increased neural activity in the PrL (A) Schematic diagram showing the recording system used to obtain scratching signals upon exposure to a distracting stimulus. (B) Flow diagram of the distraction manipulations and itch-induced scratching behaviors tests. (C–H) The distracting stimulus significantly impaired scratching behaviors during acute itch induced by intradermal injection of 5-HT (C, nape; F, cheek), compound 48/80 (D, nape; G, cheek), or chloroquine (E, nape; H, cheek). n = 8–10 rats in each group. (I) Flow diagram of fiber photometry experiments. D.S., distracting stimulus. (J) Left: Diagram showing the injection of rAAV 2/9-hSyn-jGCaMP7s or rAAV 2/9-hSyn-EYFP and implantation of an optical fiber into the right PrL of rats. Right: Representative histological images. (K) Left: Peri-event plots of the average calcium transients of global PrL neurons in response to the distracting stimulus. The thick lines and shaded regions indicate the mean ± SEM. Right: Quantification of average jGCaMP7s and EYFP fluorescence changes upon exposure to the distracting stimulus (from 0 s to 5 s). n = 5 rats in each group. The data are presented as the mean ± SEM and have a normal distribution. Two-tailed Unpaired Student’s *t* test; ∗∗p < 0. 01, ∗∗∗p < 0.001.

After verifying the reliability of the Tet-Off system, we specifically labeled itch-responsive neurons to observe or manipulate their activity. First, we bilaterally injected rAAV 2/9-*c*-Fos-tTA together with rAAV 2/9-TRE-tight-jGCaMP7s (or rAAV 2/9-TRE-tight-EGFP as a control) into the PrL (Figure 4B). After virus injection, the rats were fed with a Dox diet to prevent the expression of jGCaMP7s or EGFP. Four weeks after virus injection, Dox diet feeding was suspended for three days, and itch-responsive neurons were labeled with jGCaMP7s or EGFP by intradermally injecting 5-HT into the cheek. Dox diet feeding was resumed immediately after labeling, ensuring no interference from other factors (Figure 4A). Further histological staining of brain sections confirmed that jGCaMP7s expression was successfully induced (Figure 4B). Then, we performed *in vivo* calcium imaging studies in which fluorescent signals were recorded with fiber photometry after intradermal injection of pruritogens (5-HT or chloroquine) into the cheek. As expected, the Ca^2+^ transients of itch-responsive neurons in the PrL were significantly increased during pruritogen-evoked scratching beginning at the onset of scratching behaviors (Figure 4, Figure 5C and 5D).

The fiber photometry experiments described above showed that the activity of itch-responsive neurons was significantly increased during pruritogen-evoked scratching, suggesting a potential role for itch-responsive neurons in itch processing. We further manipulated itch-responsive neurons in the PrL. As in the previous experiment, we selectively labeled and inhibited itch-responsive neurons using the Tet-Off system and pharmacogenetic strategies (Figure 4E). The rAAV 2/9-*c*-Fos-tTA in combination with rAAV 2/9-TRE-tight-hM4Di-mCherry (or rAAV 2/9-TRE-tight-mCherry) was bilaterally injected into the PrL. After pruritogen administration, hM4Di-mCherry expression in the PrL was observed (Figure 4F). The efficacy of hM4Di-mediated inhibition has been verified in our recent study.^28^ Then, we tested the effect of pharmacogenetic inhibition of itch-responsive neurons on scratching behaviors evoked by intradermal injection of pruritogens (5-HT, compound 48/80, or chloroquine) into the cheek or nape of the neck. Pharmacogenetic inhibition of itch-responsive neurons expressing hM4Di-mCherry obviously decreased the number of scratching bouts induced by 5-HT, compound 48/80, or chloroquine (Figures 4H–4K and S3B–3E). However, rats expressing mCherry did not show any significant changes in itch processing (Figures 4H–4K and S3B–S3E). In addition, we reassessed 5-HT-evoked scratching behaviors in the absence of CNO at the end of the experiment. As expected, there were no significant differences in the number of evoked scratching bouts between the hM4Di group and the control group under these conditions (Figures 4L and S3F). These results suggest that PrL itch-responsive neurons are involved in pruritogen-induced scratching.

### A distracting stimulus significantly disrupted itch-induced scratching behaviors and increased neural activity in the prelimbic cortex

According to the results obtained thus far, it is obvious that the PrL is involved in the regulation of itch processing and that there is a population of itch-responsive neurons in the PrL that modulate itch. Moreover, previous studies have shown that the PrL is closely related to attention regulation.^22^^,^^23^^,^^24^ Therefore, our recent data combined with previous findings drive us to ask whether the involvement of the PrL in itch processing is attributable to the participation of PrL in attention modulation. To answer this question, we established an attentional bias model in which rats focused more attention on a distracting stimulus than on an itch stimulus by using a flashing light as the distracting stimulus (Figures 5A and 5B).^16^^,^^37^ The number of scratching bouts was significantly decreased in rats exposed to a distracting stimulus compared with control rats not exposed to a distracting stimulus after intradermal injection of pruritogens (Figures 5C–5H and S4). In addition, *in vivo* calcium imaging studies revealed that a distracting stimulus alone also significantly enhanced the calcium signals of PrL neurons (Figures 5J and 5K). These findings indicate that attention plays a pivotal role in itch processing and that the PrL regulates itch-scratching cycle, possibly by modulating attention; i.e., the PrL probably plays a vital role in modulating the attentional processing of itch.

### Prelimbic cortex attention-related neurons were essential for itch-scratching cycle

**Figure 6:**
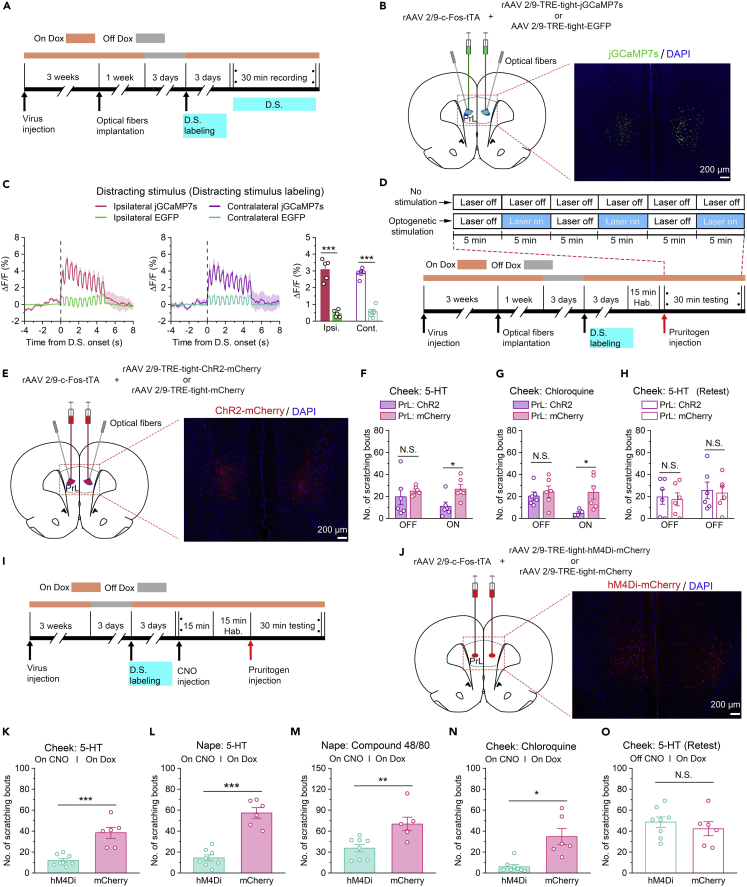
Attention-related neurons in the PrL positively regulated itch processing (A) Flow diagram of the basic experimental timeline for attention-related neuron labeling and fiber photometry. D.S., distracting stimulus. (B) Left: Schematic showing bilateral injection of rAAV 2/9-*c*-Fos-tTA and rAAV 2/9-TRE-tight-jGCaMP7s (or rAAV 2/9-TRE-tight-EGFP) and implantation of optical fibers into the PrL of rats. Right: Representative histological images. (C) Left and middle: Mean fluorescent signal of PrL attention-related neurons in response to the distracting stimulus. The thick lines and shaded regions indicate the mean ± SEM. Right: Quantification of average jGCaMP7s and EGFP fluorescence changes upon exposure to the distracting stimulus (from 0 s to 5 s). n = 5 rats. (D) Flow diagram of the basic experimental timeline for attention-related neuron labeling and optogenetic activation manipulations. D.S., distracting stimulus. (E) Left: Schematic showing injection of rAAV 2/9-*c*-Fos-tTA mixed with rAAV 2/9-TRE-tight-ChR2-mCherry or rAAV 2/9-TRE-tight-mCherry and implantation of optical fibers in the bilateral PrL of rats. Right: Representative histological images. (F and G) Optogenetic activation of attention-related neurons in the PrL significantly suppressed scratching behaviors during acute itch induced by intradermal injection of 5-HT (cheek, F) or chloroquine (cheek, G). n = 6 rats in each group. (H) Rats fed with a Dox diet were injected with 5-HT in the absence of illumination to reassess the decrease in the number of scratching bouts resulting from optogenetic activation. n = 6 rats in each group. (I) Flow diagram of the basic experimental timeline for attention-related neuron labeling and chemogenetic inhibition manipulations. D.S., distracting stimulus. (J) Left: Schematic showing bilateral injection of rAAV 2/9-*c*-Fos-tTA and rAAV 2/9-TRE-tight-hM4Di-mCherry (or rAAV 2/9-TRE-tight-mCherry) into the PrL of rats. Right: Representative histological images. (K–N) Chemogenetic inhibition of attention-related neurons significantly impaired scratching behaviors during acute itch induced by intradermal injection of 5-HT (K, cheek; L, nape), compound 48/80 (M, nape) or chloroquine (N, cheek). n = 8 rats in each group. (O) Rats fed with a Dox diet were injected with 5-HT in the absence of CNO to reassess the impairment of itch-induced scratching behaviors resulting from chemogenetic inhibition. n = 8 rats in each group. The data are presented as the mean ± SEM and have a normal distribution. Two-tailed Unpaired Student’s *t* test; N.S., not significant, ∗p < 0.05, ∗∗p < 0. 01, ∗∗∗p < 0.001.

Next, we coupled the promoters of c-Fos and TRE through a method similar to that described above (Figure 6A) and labeled attention-related neurons in the PrL that specifically contributed to attentional bias and manipulate their activity. First, we bilaterally injected rAAV 2/9-*c*-Fos-tTA together with rAAV 2/9-TRE-tight-jGCaMP7s (or rAAV 2/9-TRE-tight-EGFP as a control) into the PrL Figure 6B). Dox diet feeding was stopped for three days to label attention-related neurons with jGCaMP7s or EGFP, and the rats were exposed to a distracting stimulus. Fiber photometry studies showed that the activity of attention-related neurons in the PrL was significantly increased after exposure to a distracting stimulus (Figure 6C), confirming the existence of attention-specific neurons in the PrL.

Therefore, to explore the roles of attention-related neurons in the PrL in regulating itch processing, we used an optogenetic technique to selectively activate these attention neurons (Figure 6D). We bilaterally coinjected rAAV 2/9-*c*-Fos-tTA and rAAV 2/9-TRE-tight-ChR2-mCherry (or rAAV 2/9-TRE-tight-mCherry as a control) into the PrL (Figure 6E) and implanted optical fibers into the PrL three weeks later. Four weeks after virus injection, Dox diet feeding was stopped for 3 days to label attention-related neurons with ChR2-mCherry or mCherry, and the rats were exposed to a distracting stimulus. The efficacy of ChR2-mediated activation has been verified in our recent study.^28^ During the subsequent behavioral test, laser-on (473 nm) and laser-off periods were alternated, with both the laser-on periods and the laser-off periods lasting 5 min. The results showed that there were significantly fewer total scratching bouts in the ChR2 group than that in the mCherry group during the laser-on periods, and that there was no significant difference in the number of scratching bouts during the laser-off periods (Figures 6F and 6G and S5). In this experiment, we activated the attention-related neurons in the PrL by optogenetics techniques, to inhibit the itch processing of rats. This manipulation was equivalent to the simulation of attentional bias in rats after exposure to a distracting stimulus during itch. The results showed that itch processing was significantly affected; thus, the previously observed experimental phenomenon was reproduced. Accordingly, this evidence supports the hypothesis that the PrL modulates itch-scratching cycle by regulating attentional bias during itch processing.

Similarly, to explore the roles of itch-related neurons in the PrL in regulating itch processing, we used the same method as above to selectively activate these itch-responsive neurons (Figures S6A and S6B). The results showed that the scratching behaviors were suppressed in the ChR2 group than that in mCherry group during the laser-on periods, and that there was no significant difference in the laser-off periods (Figures S6D and S6E). Rats fed with a Dox diet were injected with 5-HT in the absence of illumination to reassess the decrease in the number of scratching bouts resulting from optogenetic activation. As expected, there was no significant difference between the ChR2 group and the mCherry group under these conditions (Figure S6F). We analyzed this phenomenon because, similar to light interference, activating these itch-responsive neurons occupied the attention channels of rats, thus reducing the scratching behaviors.

Next, we further confirmed our hypothesis by using chemogenetic techniques to investigate the role of attention-related neurons in the PrL in itch processing. The hybrid virus rAAV 2/9-*c*-Fos-tTA in combination with rAAV 2/9-TRE-tight-hM4Di-mCherry (or rAAV 2/9-TRE-tight-mCherry as control) was injected bilaterally into the PrL (Figure 6J). Three days after stopping Dox diet feeding, attention-related neurons activated by a distracting stimulus were labeled with hM4Di-mCherry or mCherry (Figure 6I). The results showed that the inhibition of PrL attention-related neurons also significantly reduced the total number of scratching bouts (Figures 6K–6N and S7A–S7D). In addition, we reassessed 5-HT-evoked scratching behaviors in the absence of CNO. As expected, there was no statistically significant difference in the number of scratching bouts between the hM4Di-expressing group and the control group under these conditions (Figures 6O and S7E). These findings suggest that attention focused on itch can be disrupted by selectively suppressing attention-related neurons in the PrL, thus inhibiting itch-induced scratching behaviors.

### Itch-responsive neurons and attention-related neurons obviously overlapped in the prelimbic cortex

**Figure 7:**
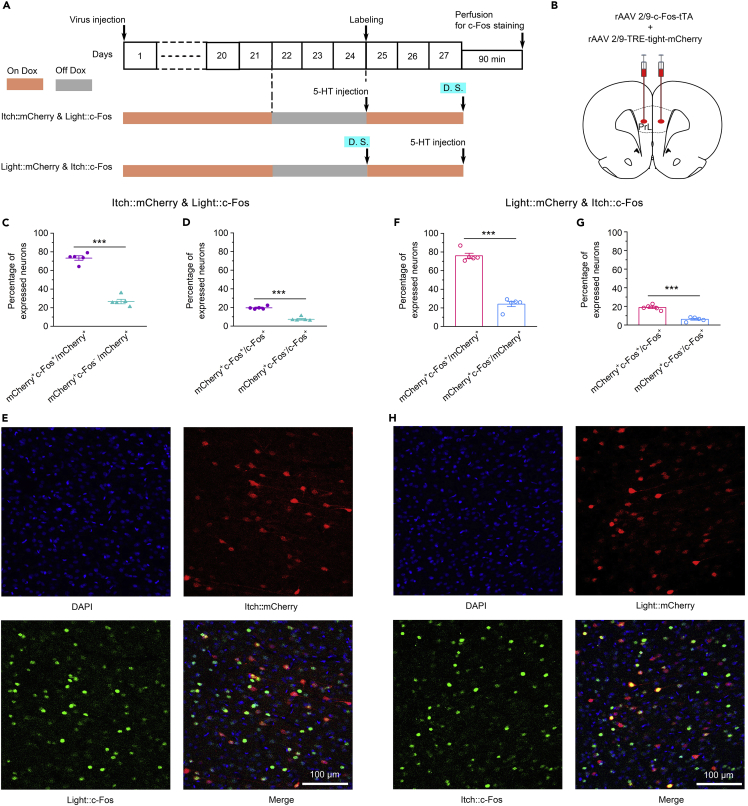
High overlap between itch-responsive neurons and attention-related neurons in the PrL (A) Flow diagram of the basic experimental timeline for itch-responsive neuron and attention-related neuron labeling. D.S., distracting stimulus. (B) Schematic showing bilateral injection of rAAV 2/9-*c*-Fos-tTA and rAAV 2/9-TRE-tight-mCherry into the PrL. (C and D) In the itch:mCherry and light:c-Fos expression group, the percentage of double-positive (mCherry^+^c-Fos^+^) neurons relative to mCherry^+^ neurons (C) or c-Fos^+^ neurons (D) was significantly higher than the percentage of mCherry^+^c-Fos^-^ neurons. n = 5 rats in each group. (E) Representative images of distracting stimulus-induced endogenous c-Fos expression in the PrL of rats with mCherry^+^ itch-responsive neurons (itch:mCherry). (F and G) In the light:mCherry and itch:c-Fos expression group, the percentage of double-positive (mCherry^+^c-Fos^+^) neurons relative to mCherry^+^ neurons (F) or c-Fos^+^ neurons (G) was significantly higher than the percentage of mCherry^+^c-Fos^-^ neurons. n = 5 rats in each group. (H) Representative images of endogenous c-Fos expression elicited by 5-HT-induced itch in the PrL of rats with mCherry^+^ attention-related neurons (light:mCherry). The data are presented as the mean ± SEM and have a normal distribution. Two-tailed Unpaired Student’s *t* test; ∗∗∗p < 0.001.

Hence, to further explore the mechanism of the PrL in the regulation of itch processing via attentional bias, we continued to investigate the relationship between itch-responsive neurons and attention-related neurons in the PrL. To simultaneously label two subpopulations in the PrL, we bilaterally injected rAAV 2/9-cfos-tTA and rAAV 2/9-TRE-tight-mCherry into the PrL (Figure 7B) and fed the rats with a Dox diet for 21 days. Dox diet feeding was then stopped for 3 days to allow expression of the mCherry, and the rats were either intradermally injected with 5-HT into the cheek or exposed to a distracting stimulus on the 24th day to allow labeling of itch-responsive neurons (i.e., itch:mCherry) or attention-related neurons (i.e., light:mCherry), respectively. Three days after labeling, the rats in the two groups were either exposed to a distracting stimulus or intradermally injected with 5-HT into the left cheek. Ninety minutes later, the brains were collected and stained for endogenous c-Fos (Figure 7A). We were able to simultaneously observe the distribution of itch-responsive neurons and attention-related neurons by assessing mCherry fluorescence and immunofluorescence staining of endogenous c-Fos.

The histological results showed that in rats expressing itch:mCherry and light:c-Fos, up to 73.29% of mCherry^+^ neurons activated by itch overlapped with c-Fos^+^ neurons activated by a distracting stimulus (Figures 7C and 7E). Furthermore, the percentage of double-positive (mCherry^+^c-Fos^+^) neurons relative to mCherry^+^ neurons or c-Fos^+^ neurons was significantly higher than the percentage of mCherry^+^c-Fos^-^ neurons (Figures 7C and 7D). Similarly, in the light:mCherry and itch:c-Fos group, 75.91% of mCherry^+^ neurons activated by a distracting stimulus overlapped with c-Fos^+^ neurons activated by itch (Figures 7F and 7H). Moreover, the percentage of mCherry^+^c-Fos^+^ neurons relative to mCherry^+^ neurons or c-Fos^+^ neurons was significantly higher than the percentage of mCherry^+^c-Fos^-^ neurons (Figures 7F and 7G). Together, these findings indicate that there is an obvious overlap between itch-responsive neurons and attention-related neurons in the PrL at the histological level.

### Functional activation of itch-responsive neurons in the prelimbic cortex was interchangeable with that of attention-related neurons

**Figure 8:**
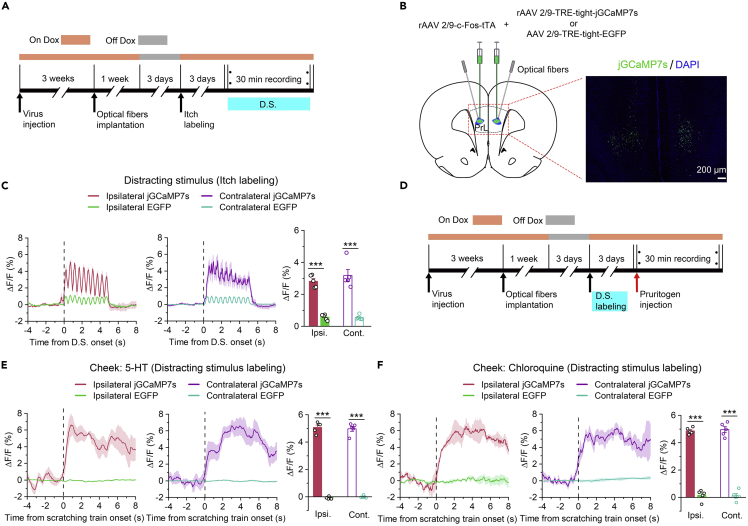
The functional activation of itch-responsive neurons in the PrL was interchangeable with the functional activation of attention-related neurons (A) Flow diagram of the basic experimental timeline for itch-responsive neuron labeling and fiber photometry. D.S., distracting stimulus. (B) Left: Schematic showing injection of rAAV 2/9-*c*-Fos-tTA and rAAV 2/9-TRE-tight-jGCaMP7s (or rAAV 2/9-TRE-tight-EGFP) and implantation of optical fibers in the bilateral PrL of rats. Right: representative histological images. (C) Left and middle: Mean fluorescent signal of PrL itch-responsive neurons in response to the distracting stimulus. The thick lines and shaded regions indicate the mean ± SEM. Right: Quantification of average jGCaMP7s and EGFP fluorescence changes upon exposure to the distracting stimulus (from 0 s to 5 s). n = 5 rats in each group. (D) Flow diagram of the basic experimental timeline for attention-related neuron labeling and fiber photometry. D.S., distracting stimulus. (E and F) Left and middle: Mean fluorescent signal of PrL attention-related neurons in response to the itch scratching induced by intradermal injection of 5-HT (E) or chloroquine (F). The thick lines and shaded regions indicate the mean ± SEM. Right: Quantification of average jGCaMP7s and EGFP fluorescence changes upon exposure to the distracting stimulus (from 0 s to 6 s). n = 5 rats in each group. The data are presented as the mean ± SEM and have a normal distribution. Two-tailed Unpaired Student’s *t* test; ∗∗∗p < 0.001.

We next examined whether the functional activation of itch-responsive neurons in the PrL is interchangeable with that of attention-related neurons. We bilaterally injected rAAV 2/9-*c*-Fos-tTA and rAAV 2/9-TRE-tight-jGCaMP7s (or rAAV 2/9-TRE-tight-EGFP as a control) into the PrL (Figure 8B). We first labeled itch-responsive neurons in the PrL with jGCaMP7s or EGFP (Figure 8A). As we expected, we found that the activity of itch-responsive neurons was significantly increased upon exposure to a distracting stimulus (Figure 8C). We also used a distracting stimulus to label attention-related neurons in the PrL with jGCaMP7s or EGFP (Figure 8D). Consistently, the activity of attention-related neurons was obviously increased during itch processing (Figures 8E and 8F). Fiber photometry experiments also confirmed that a distracting stimulus can activate itch-responsive neurons, while itch can also activate attention-related neurons, indicating that the functional activation of the two subpopulations is interchangeable at the cellular activity level.

## Discussion

Itch is a complex aversive sensation that elicits a strong urge to scratch, and scratching suppresses the itching sensation, creating an itch-scratching cycle.^5^^,^^38^ Previous studies have clearly elucidated the mechanisms of itch at the level of primary afferent fibers and the spinal cord.^39^^,^^40^ However, there is little knowledge about the brain cells and neural circuits underlying the regulation of different components of itch. In this study, we demonstrated the important regulatory effect of PrL on the attentional processing of itch. First, we showed that the PrL was associated with itch processing using fiber photometry, chemogenetic, optogenetic manipulation, and further behavioral experiments demonstrated the existence of itch-responsive neurons that regulate itch processing in the PrL. Fortunately, we found that the attentional bias caused by a distracting stimulus also significantly affected the itch-induced scratching behaviors of rats. Furthermore, the calcium signals of PrL neurons were significantly increased upon exposure to a distracting stimulus, suggesting that the regulatory effect of the PrL on itch may be closely related to attentional bias. Then, to investigate the essential neural mechanisms underlying itch processing in the PrL, we optogenetically activated attention-related neurons in the PrL to simulate the effect of a distracting stimulus and found that the itch-induced scratching behaviors of rats were significantly inhibited. Furthermore, chemogenetic inhibition of attention-related neurons effectively inhibited itch-induced scratching behaviors. Thus, these findings suggest that the two neuronal subsets are interchangeable in the regulation of itch processing. Additionally, histological experiments showed that itch-responsive neurons and attention-related neurons largely overlapped in the PrL. The fiber photometry results showed that the functional activity of the two subpopulations was also interchangeable at the cellular activity level. Collectively, these results strongly support the conclusion that the PrL plays a critical role in modulating the attentional processing of itch.

The sensation of itch originates from a pattern of activity changes in various regions of the brain.^39^ The same skin irritation can induce very different itch perceptions depending on the state of consciousness and attention.^16^ Several clinical psychological experiments in humans have confirmed that sensitivity to pruritus and pain is affected by concentration on physical sensation.^19^^,^^41^ In addition, frontal cortical regions, especially the dorsolateral prefrontal cortex, have been confirmed to be critical for attention allocation.^42^^,^^43^ In the present study, we established an attentional bias model by using a flashing light as a distracting stimulus and confirmed that PrL plays an important role in the modulation of the attentional processing of itch (Graphical abstract).

It is important to note that during fiber photometry recording, the calcium fluorescence intensity of itch-responsive neurons (e.g., Figure 2C) and attention-related neurons (e.g., Figure 6E) was much lower than that of global neurons (e.g., Figure 1H) during the same pruritogen (e.g., 5-HT)-induced scratching trains. In contrast, the calcium fluorescence intensity of these global neurons was approximately equal when the rats were injected with different pruritogens (Figures 1E–1J). Moreover, itch-responsive neurons displayed approximately equal increases in calcium fluorescence intensity upon the injection of different pruritogens (Figures 4C and 4D) and upon exposure to a distracting stimulus (Figure 8C). Consistently, the calcium fluorescence intensity of attention-related neurons was also approximately equal upon exposure to a distracting stimulus (Figure 6A) and administration of different pruritogens (Figures 8E–8F). The reason for these interesting results is that the itch-responsive neurons and attention-related neurons labeled by the Tet-Off system were able to express jGCaMP7s for a limited period of time (approximately 30 min), while global neurons were able to continuously express jGCaMP7s for approximately four weeks.^44^ Therefore, the level of jGCaMP7s in each itch-responsive neuron and attention-related neuron was significantly lower than that in the global neurons. The differential expression of jGCaMP7s can be observed in the histological images (e.g., Figure 1, Figure 4D and 4B). Moreover, the level of jGCaMP7s directly determines the calcium fluorescence intensity in response to itch-induced scratching behaviors and a distracting stimulus. Thus, the calcium fluorescence intensity of itch-responsive neurons and attention-related neurons was much lower than that of global neurons. These results strongly support our hypothesis since itch-responsive neurons and attention-related neurons had similar calcium fluorescence intensities upon the injection of different pruritogens and upon exposure to a distracting stimulus.

It is worth noting that the percentage of endogenous c-Fos^+^ neurons labeled by immunofluorescence staining was always higher than that of mCherry^+^ neurons labeled by the Tet-Off system. The different mechanisms of the two labeling techniques may account for this discrepancy. In the Tet-Off system, expression of the c-Fos protein in activated neurons enables the c-Fos promotor (rAAV 2/9-*c*-Fos-tTA) to induce the expression of the second virus (rAAV 2/9-TRE-tight-mCherry). It is possible that the lower expression of c-Fos observed in labeled neurons expressing the Tet-Off system than in neurons subjected to immunofluorescence staining resulted from these two steps. In addition, c-Fos immunofluorescence staining is a very sensitive method for examining the neural activity of neurons and thus may cause false positive results and increase the percentage of endogenous c-Fos^+^ neurons.^45^ Correspondingly, to eliminate the influence of these differences, we performed two corresponding histological experiments in which we used both the Tet-Off system and c-Fos immunofluorescence staining to label itch-responsive neurons and attention-related neurons. Both results showed that the percentage of double-positive (mCherry^+^c-Fos^+^) neurons relative to mCherry^+^ neurons or c-Fos^+^ neurons was significantly higher than the percentage of mCherry^+^c-Fos^-^ neurons. Therefore, these results support the notion that itch-responsive neurons and attention-related neurons overlap significantly at the histological level.

Notably, we only indicated that another outside stimulus distracts the attention from itch, which in turn reduces the itch. However, the present findings did not indicate that all attention bias reduces itch. Some attentional stimuli may induce the urge to scratch. For example, recent studies have shown that visual attention to scratching mice induces contagious itch.^46^^,^^47^ However, the biggest difference in their studies is that observer mice are not injected with pruritogens, still scratched by paying attention to conspecific scratching. In our study, the animals pay attention to distracting stimulus from itch. Additionally, they have reported that Gastrin-releasing peptide and its receptor in the suprachiasmatic nucleus of the hypothalamus mediates contagious itch behavior,^46^ and that suprachiasmatic nucleus-projecting intrinsically photosensitive retina ganglion cells are necessary and sufficient for contagious itch behaviors.^47^ Our study showed that two distinct stimuli which trigger an attentional bias activate overlapping populations of neuron in the PrL and that the PrL plays an important role in the modulation of the attentional distraction from itch. Thus, our recent data combined with these findings suggest that attentional processing may engage the modulation of multiple brain regions.

At present, despite recent progress in our understanding of the neuronal basis of itch in the peripheral and central nervous systems,^9^^,^^13^^,^^14^ many neuronal and circuit mechanisms underlying itch modulation remain poorly understood. Because a full understanding of the mechanisms underlying itch is lacking, current treatments for chronic itch are largely ineffective, which seriously affects the physical and mental health of patients. Studying the role of attention in itch may unravel the relationship between attentional processing and somatosensory sensations, and the findings may contribute to the improvement of itch treatment in the long term.^16^ Our study reveals the regulatory mechanism of the attention system involved in itch processing, which may provide us with exciting insights into the neuronal mechanisms underlying itch and may provide a new perspective for the in-depth study of effective treatments for pruritus.

### Limitations of the study

This study also has some limitations. For example, attention-related experiments conducted on humans can be evaluated by human reaction time or actions, but rats cannot make clear responses or have relevant models to evaluate. In addition, the rats may gradually adapt to the distracting stimulus without changing attention from itch to flashing light. Besides, although we have proved that the itch-responsive neurons and attention-related neurons are highly overlapping in animal behavior, histology and cell functional activity, the specific overlapping mechanism still needs further research in future studies. At the same time, the functional differences between the non-overlapping regions of these two neuronal subsets are also worth further studies.

## STAR★Methods

### Key resources table

**Table:** 

REAGENT or RESOURCE	SOURCE	IDENTIFIER
**Antibodies**		

Rabbit anti-c-Fos	Santa Cruz	Cat# sc-52
Rabbit anti-c-Fos	Synaptic Systems	Cat# 226003
Goat anti-rabbit conjugated to Alexa Fluor™ 488	Invitrogen	Cat# A11034

**Bacterial and virus strains**		

rAAV 2/9-hSyn-jGCaMP7s	OBiO Technology, China	N/A
rAAV 2/9-hSyn-EYFP	OBiO Technology, China	N/A
rAAV 2/9-hSyn-hM4Di-mCherry	OBiO Technology, China	N/A
rAAV 2/9-hSyn-mCherry	OBiO Technology, China	N/A
rAAV 2/9-hSyn-eNpHR3.0-EYFP	OBiO Technology, China	N/A
rAAV 2/9-c-Fos-tTA	OBiO Technology, China	N/A
rAAV 2/9-TRE-tight-hM4Di-mCherry	OBiO Technology, China	N/A
rAAV 2/9-TRE-tight-mCherry	OBiO Technology, China	N/A
rAAV 2/9-TRE-tight-jGCaMP7s	OBiO Technology, China	N/A
rAAV 2/9-TRE-tight-EGFP	OBiO Technology, China	N/A
rAAV 2/9-TRE-tight-ChR2-mCherry	OBiO Technology, China	N/A

**Chemicals, peptides, and recombinant proteins**		

Compound 48/80	Sigma-Aldrich	Cat# C2313
5-HT	Sigma-Aldrich	Cat# H9523
Chloroquine	Sigma-Aldrich	Cat# C6628
Clozapine-N-oxide, CNO	MCE	Cat# HY-17366
Paraformaldehyde	Sigma-Aldrich	Cat# P6148
DAPI	Sigma-Aldrich	Cat# D9542

**Experimental models: Organisms/strains**		

Sprague-Dawley (SD) rats	Laboratory Animal Center, Army Medical University, China	N/A

### Resource availability

#### Lead contact

Further information and requests for resources and reagents should be directed to and will be fulfilled by the lead contact, Guang-Yan Wu (wgy009@163.com).

#### Materials availability

This study did not generate new unique reagents.

### Experimental model and subject details

#### Animals

Adult male Sprague–Dawley (SD) rats weighing 300–350 g (3–4 months) at the time of virus injection were individually housed in standard stainless steel cages on a 12 h light/dark cycle at 21–24 °C with *ad libitum* access to food and water. Behavioral experiments were performed during the light phase at 25 ± 1 °C. All animal procedures were approved by the Animal Care Committee of the Army Medical University and were performed in accordance with the principles outlined in the National Institutes of Health Guide for the Care and Use of Laboratory Animals.

### Method details

#### Virus injection

Rats were deeply anesthetized and then fixed in a stereotaxic apparatus (Model 942, David Kopf Instruments, Tujunga, California, USA). The viruses were injected at a rate of 50 or 60 nL/min using a glass pipette with a tip diameter of approximately 20 μm, controlled by a stereotaxic microsyringe pump (53311, Stoelting, USA), or directly controlled by a programmable nanoliter injector (Nanoject III, Drummond Scientific, USA). After the injection, the needle was left in place for 5 min and then slowly withdrawn. Finally, the incision was closed with sutures.

For fiber photometry recording of PrL global neuron activity during itch processing, the rats were microinjected with 150 nL of rAAV 2/9-hSyn-jGCaMP7s (titer: 5.75 × 10^12^ vg/mL) or rAAV 2/9-hSyn-EYFP as a control (titer: 4.89 × 10^12^ vg/mL) into the right PrL at the following coordinates: +3.10 mm anteroposterior (AP), 0.70 mm mediolateral (ML), and −4.30 mm dorsoventral (DV; Figure 1D).

Following chemogenetic inhibition of PrL global neurons during itch-induced scratching behaviors, rats were bilaterally microinjected with rAAV 2/9-hSyn-hM4Di-mCherry (titer: 6.96 × 10^12^ vg/mL) into the PrL (+3.10 mm AP, ±0.70 mm ML, −4.30 mm DV) in a volume of 150 nL for each side or rAAV 2/9-hSyn-mCherry virus (titer: 6.25 × 10^12^ vg/mL) as a control (Figure 2B).

For optogenetic inhibition of PrL global neurons, neurons expressing eNpHR3.0 were inhibited by 593-nm laser illumination. Rats were bilaterally microinjected with 150 nL of rAAV 2/9-hSyn-eNpHR3.0-EYFP (titer: 1.73 × 10^12^ vg/mL) or rAAV 2/9-hSyn-EYFP as a control (titer: 4.89 × 10^12^ vg/mL) into the PrL (+3.10 mm AP, ±0.70 mm ML, −4.30 mm DV; Figure 3B).

To test the reliability of the Tet-Off system, rats were bilaterally microinjected with rAAV 2/9-*c*-Fos-tTA (titer: 2.58× 10^12^ vg/mL) and rAAV 2/9-TRE-tight-mCherry (titer: 2.25 × 10^12^ vg/mL) into the PrL (+3.10 mm AP, ±0.70 mm ML, −4.30 mm DV) in a volume of 150 nL for each side. The expression of virus under different conditions was analyzed by histological methods (Figure S2C).

For labeling and fiber photometry recording of itch-responsive neurons or attention-related neurons in the PrL, we bilaterally microinjected rAAV 2/9-*c*-Fos-tTA (titer: 2.58× 10^12^ vg/mL) mixed with rAAV 2/9-TRE-tight-jGCaMP7s (titer: 2.25 × 10^12^ vg/mL) or rAAV 2/9-TRE-tight-EGFP (titer: 2.39 × 10^12^ vg/mL) as a control into the PrL (+3.10 mm AP, ±0.70 mm ML, −4.30 mm DV) in a volume of 150 nL for each side (Figure 4, Figure 6, Figure 8, 6, and 8B).

For labeling and pharmacogenetic inhibition of itch-responsive neurons or attention-related neurons in the PrL, we bilaterally microinjected 150 nL of both rAAV 2/9-*c*-Fos-tTA (titer: 2.58× 10^12^ vg/mL) and rAAV 2/9-TRE-tight-hM4Di-mCherry (titer: 2.25 × 10^12^ vg/mL) or rAAV 2/9-TRE-tight-mCherry (titer: 2.39 × 10^12^ vg/mL) in the same volume as the control into the PrL (+3.10 mm AP, ±0.70 mm ML, −4.30 mm DV; Figure 4, Figure 6F and 6J, respectively).

For labeling and optogenetic activation of itch-responsive neurons or attention-related neurons in the PrL, rats were bilaterally microinjected with 150 nL of both rAAV 2/9-*c*-Fos-tTA (titer: 2.58× 10^12^ vg/mL) and rAAV 2/9-TRE-tight-ChR2-mCherry (titer: 1.10 × 10^12^ vg/mL) or rAAV 2/9-TRE-tight-mCherry (titer: 2.39 × 10^12^ vg/mL) as a control into the PrL (+3.10 mm AP, ±0.70 mm ML, −4.30 mm DV; Figures S6 and 6E, respectively).

To evaluate the histological relationship between itch-responsive neurons and attention-related neurons in the PrL, we bilaterally microinjected 150 nL of both rAAV 2/9-*c*-Fos-tTA (titer: 2.58× 10^12^ vg/mL) and rAAV 2/9-TRE-tight-mCherry (titer: 2.39 × 10^12^ vg/mL) into the PrL (+3.10 mm AP, ±0.70 mm ML, −4.30 mm DV; Figure 7B).

#### Optical fiber implantation

Rats were deeply anesthetized and then fixed in a stereotaxic apparatus. Then, for optogenetic manipulations, optical fibers (ceramic ferrule: diameter of 2.50 mm; optical fiber: 200 μm core diameter) were bilaterally implanted into the PrL at a 15-degree angle (+3.10 mm AP, ±0.70 mm ML, −4.00 mm DV; e.g., Figure 3B). For fiber photometry recording, the optical fibers were placed 50 μm above the viral injection site in the right PrL at a 0-degree angle (+3.10 mm AP, ±0.70 mm ML, −4.25 mm DV; Figure 1D) or bilateral PrL at a 15-degree angle (+3.10 mm AP, ±0.70 mm ML, −4.25 mm DV; e.g., Figure 4B). The optical fibers were secured to the skull with dental cement in a stereotaxic apparatus 3 weeks after virus injection. After the surgery, the animals were allowed to recover for 1 week. In all experiments, the rats underwent behavior tests at least 4 weeks after the initial surgery.

#### Itch-responsive neuron labeling

To label itch-responsive neurons in the PrL, rats were fed with food containing 100 mg/kg Dox for three or four weeks after virus injection. Three or four weeks later, the Dox diet was replaced with regular food without Dox for 3 days to allow activity-dependent labeling for a window of time. Three days after stopping Dox diet feeding, 5-HT was intradermally injected into the left cheek to induce scratching behaviors and neuronal activity. Thirty minutes after 5-HT injection, the rats were returned to their home cages and fed with a Dox diet (e.g., Figures 4A and 4D).

#### Attention-related neurons labeling

To label distracting stimulus-responsive neurons in the PrL, rats were fed with food containing 100 mg/kg Dox for three or four weeks after viral injection. Then, the Dox diet was replaced with regular food without Dox for 3 days to allow activity-dependent labeling for a window of time. Three days after stopping Dox diet feeding, we used simple flashing lights (250 ms on/off for 5 s, 30 min, 8 lux) emitted by two LEDs to induce attentional bias and neuronal activity. The interstimulus interval (ISI; the interval between the onset of two consecutive flashing light trains) was 15–20 s (Figure 5B). Thirty minutes after exposure to the distracting stimulus, the rats were returned to their home cages and fed with a Dox diet.

#### Itch test

According to the experimental design, two or five days before the behavioral tests, the nape of the neck or/and cheek of each rat was shaved. For automatic assessment of scratching behaviors, a small rare-earth magnet ring (outside diameter: 11–12 mm, inside diameter: 7.8–8.8 mm, height: 2 mm) was attached to the hindlimb of each rat. The magnet rings, which were well-tolerated by awake rats, were placed daily immediately before habituation and behavioral tests and removed immediately after habituation and behavioral testing. In addition, the rats were habituated to a plastic behavioral recording box (30 ×30 ×35 cm) for 30 min for 2 consecutive days and housed in a sound-attenuating chamber prior to experimentation and data collection. The recording box was surrounded by an induction coil connected to a differential amplifier (model 3500, A-M Systems, USA) at the level of the feet (Figure S1).

During the behavioral experiments, baseline recordings were performed in the recording box for 15 min. Then, the rats were briefly removed from the recording box, and different pruritogens were intradermally injected into the cheek or nape of the neck. For the cheek itch model, rats received an intradermal injection of compound 48/80 (65.36 mM in sterile saline; C2313, Sigma-Aldrich), 5-HT (20.00 mM in sterile saline; H9523, Sigma-Aldrich), or chloroquine (77.50 mM in sterile saline; C6628, Sigma-Aldrich) into one cheek in a total volume of 50 μL. For the nape itch model, rats received an intradermal injection of compound 48/80 (65.36 mM in sterile saline), 5-HT (5.00 mM in sterile saline), or chloroquine (77.50 mM in sterile saline) into one side of the nape of the neck in a total volume of 50 μL. Scratching behaviors was recorded for 30 min after injection and was analyzed with custom-written codes in MATLAB.

#### Behavioral data analysis

To evaluate scratching behaviors, we recorded the movement of the hindlimb via the magnetic induction method, as described above. Movement of the ipsilateral hindlimb (relative to the pruritogen injection site), to which a small magnet was attached, induced robust inductive coil voltage fluctuations (defined as voltage peaks). The inductive coil voltage signals were bandpass filtered (3–100 Hz), amplified (100×) using a 16-channel differential amplifier (model 3500, A-M Systems), and acquired with a data acquisition system (Powerlab 16/35, AD Instruments) at a sampling rate of 1 kHz.

After comparative analysis of rat scratching behaviors recorded by a digital video camera and by the magnetic induction method, we found that inductive coil voltage fluctuations could be induced by both scratching and hindlimb movement (e.g., walking). However, scratching behaviors induced a cluster of voltage fluctuations, exhibiting periodic characteristics. Scratching behaviors and locomotion did not occur at the same time. Moreover, the frequency of mouse scratching behaviors was 2–25 Hz (0.04–0.5 s), consistent with a previous report, and every two voltage fluctuations corresponded to one actual scratching (rubbing) event. Thus, the frequency of voltage fluctuations induced by scratching behaviors was 4–50 Hz (0.02–0.25 s).

To detect scratching behaviors, inductive coil voltage fluctuations exceeding the threshold (300 μV) were considered movement voltage peaks. Based on the above analysis, we used the following two criteria to identify scratching behaviors signals among the movement voltage peaks of the rats: (1) an interval between two contiguous motion voltage peaks of 0.02–0.25 s (4–50 Hz), and (2) at least 4 contiguous motion voltage peaks for scratching bouts. The movement voltage peaks caused by other hindlimb movements (e.g., walking) were mostly excluded by these criteria. Given that the animals often resumed scratching after a short pause (<2.0 s), a scratching train was defined as a cluster of scratching bouts with an interval of <2.0 s. For the behavioral experiments, the number of scratches was calculated (Figure S1).

#### Pharmacogenetic manipulations

For *in vivo* chemogenetic inhibition, rats expressing hM4Di-mCherry or mCherry as a control were injected (i.p.) with 4 mg/kg CNO (diluted with 5% DMSO and saline; HY-17366, MCE), and behaviors were assessed 30 min after CNO injection.

#### Optogenetic manipulations

For *in vivo* optogenetic suppression, rats expressing eNpHR3.0-EYFP or EYFP were exposed to a 30-min rest–stimulation illumination cycle (5 min of laser off–5 min of laser on, repeated 3 times; Figure 3A) using a 593-nm laser controlled by a pulse stimulator (Master-9) immediately after pruritogen injection. A constant illumination (593 nm, ∼5 mW) was delivered bilaterally during the laser on period.

For *in vivo* optogenetic activation, rats expressing ChR2-mCherry or mCherry were exposed to a 30-min rest–stimulation illumination cycle (5 min of laser off–5 min of laser on, repeated 3 times) using a 473-nm laser controlled by a pulse stimulator (Master-9) immediately after exposure to a distracting stimulus. A pulse illumination (473 nm, ∼5 mW, 20 Hz, 15 ms) was delivered bilaterally during the laser on period (Figure 6D). In addition, for *in vivo* optogenetic activation during the balance beam test, the rats were exposed to constant laser illumination (473 nm, ∼5 mW, 20 Hz, 15 ms) immediately after the start of the test.

#### Distraction manipulations

The rats were placed in a plastic behavioral recording box (30 ×30 ×35 cm) and housed in a sound-attenuating chamber during the distracting stimulus experiments. The distracting stimulus consisted of flashing white lights (250 ms on/off for 5 s, 30 min, 8 lux) emitted from two LEDs. The ISI was 10–20 s (Figures 5A and 5B).

#### Fiber photometry

Fluorescence signals from the PrL were recorded by a fiber photometry recording system (INPER-C1-3C, Inper, China) at 100 Hz for 30 min after injection of pruritogens (e.g., Figure 1E) or upon exposure to a distracting stimulus (e.g., Figure 5K). Fluorescence signals were provided by a 488-nm laser. Moreover, a 405-nm laser was concurrently used to isolate the movement-corrected signals from the channel. The laser power at the tip of the optical fiber was adjusted to a low level (20–40 μW) to minimize bleaching of the jGCaMP7s and EYFP (or EGFP) signals.

The fiber photometry data were analyzed using the analysis software of the fiber photometry recording system. The fluorescence values during each scratching train were derived. The change in the fluorescence value (ΔF/F) was calculated by the formula (F-F0)/F0, where F refers to the fluorescence value at each time point (−4 to 8 s relative to the scratching train or distracting stimulus (flashing light) onset) and F0 refers to the median fluorescence value during the baseline period (−4 to −2 s relative to the scratching train onset; −4 to 0 s relative to the distracting stimulus onset). To visualize the fluorescence change, the ΔF/F values are presented with plots, with the shaded regions indicating the SEM. To statistically analyze the change in fluorescence values across scratching trains or distracting stimulus exposures, the mean ΔF/F was defined as the average ΔF/F value during the analysis period (0–6 s relative to the scratching train onset; 0 to 5 s relative to the distracting stimulus onset; e.g., Figure 1, Figure 5E and 5K).

#### Immunohistochemistry

To detect induction of c-Fos expression in the PrL by pruritogens, rats were intradermally injected with 5-HT (5 mM or 20 mM) or saline into one side of the nape of the neck or one cheek in a total volume of 50 μL, and their brains were collected 90 min later. Moreover, to examine endogenous c-Fos expression in the PrL induced by a distracting stimulus, rats were exposed to a distracting stimulus, i.e., flashing white lights (250 ms on/off for 5 s, 30 min, ISI 10–20 s, 8 lux) emitted from two LEDs, and their brains were also collected 90 min later.

Immunohistochemistry procedure is as follows^28^^,^^48^^,^^49^: rats were anesthetized and perfused transcardially with physiological saline followed by cold 4% paraformaldehyde (PFA; prepared in 0.1 M phosphate buffer, pH 7.4). The brains were removed from the skull and stored in 4% PFA at 4 °C for 24 h and then transferred to 30% sucrose solution at 4 °C for 48 h. Coronal sections (30 μm thick) were cut on a freezing microtome (CM3050 S, Leica, Germany) and collected in cold PBS (PBS, 0.01 M, pH 7.4). For immunostaining, each slice was placed in PBST (PBS +0.3% Triton X-100) containing 2% normal BSA for 1 h and then incubated with primary antibody at 4 °C for 24 h (rabbit anti-*c*-Fos 1:100, sc-52, Santa Cruz; rabbit anti-*c*-Fos 1:500, 226003, Synaptic Systems). The slices were then washed 3 times for 10 min each in PBST and incubated for 2 h with secondary antibody (goat anti-rabbit conjugated to Alexa Fluor 488 1:500, A11034, Invitrogen). The slices were washed with PBST (once, 10 min), incubated for 10 min with DAPI (1:2000, D9542, Sigma–Aldrich), washed 3 more times for 10 min each in PBST, mounted on microscope slides and coverslipped. Images were acquired using a Carl Zeiss LSM 780/LSM 800 laser scanning microscope (Germany) or Olympus VS120-S5/VS200 virtual slide system (Japan).

#### Histology

After the behavioral and fiber photometry experiments, rats were anesthetized and perfused transcardially with physiological saline followed by cold 4% PFA (prepared in 0.1 M phosphate buffer, pH 7.4). The brains were removed from the skull and placed in 4% PFA at 4 °C for 24 h before sectioning and then transferred to 30% sucrose solution at 4 °C for 48 h. Coronal sections (30 μm thick) were cut on a freezing microtome (CM3050 S, Leica) and collected in cold PBS (PBS, 0.01 M, pH 7.4). The slices were washed with PBST (once, 10 min), incubated for 10 min with DAPI (1:2000, D9542, Sigma–Aldrich), washed 3 more times for 10 min each in PBST, mounted on microscope slides and coverslipped. The images were acquired using an Olympus BX53F fluorescence microscope (Japan) or Olympus VS120-S5/VS200 virtual slide system.

### Quantification and statistical analysis

All the data are expressed as the mean ± SEM. Statistical significance was determined by two-tailed unpaired Student’s *t* test, one-way ANOVA followed by the LSD post hoc test, or two-way repeated measures ANOVA followed by the separate one-way ANOVA using SPSS software for Windows (v. 25.0). A value of p < 0.05 was considered statistically significant. N.S., not significant, ∗p < 0.05, ∗∗p < 0. 01, ∗∗∗p < 0.001.

## Data Availability

• The original data reported in this paper is available from the lead contact upon request. • This paper does not report original code. • Additional information related to this study is available from the lead contact upon request.
