# Supplementary material for: The prelimbic cortex regulates itch processing by controlling attentional bias
Source: iScience. 2022 Dec 17;26(1):105829. doi: 10.1016/j.isci.2022.105829 (PMC9816985; doi:10.1016/j.isci.2022.105829)
Supplement: Document S1. Figures S1–S — 7 [file mmc1.pdf]

iScience, Volume *26*

## Supplemental information

# The prelimbic cortex regulates itch processing by controlling attentional bias

**Guang-Yan Wu, Xiao-Xia Zheng, Shan-Lan Zhao, Yi Wang, Shan Jiang, Yi-Song Wang, Yi-Lun Yi, Juan Yao, Hui-Zhong Wen, Ju Liu, Hong-Li Li, and Jian-Feng Sui**

# Supplemental information

# The prelimbic cortex regulates itch processing by controlling attentional bias

Guang-Yan Wu, Xiao-Xia Zheng, Shan-Lan Zhao, Yi Wang, Shan Jiang, Yi-Song Wang, Yi-Lun Yi, Juan Yao, Hui-Zhong Wen, Ju Liu, Hong-Li Li, and Jian-Feng Sui

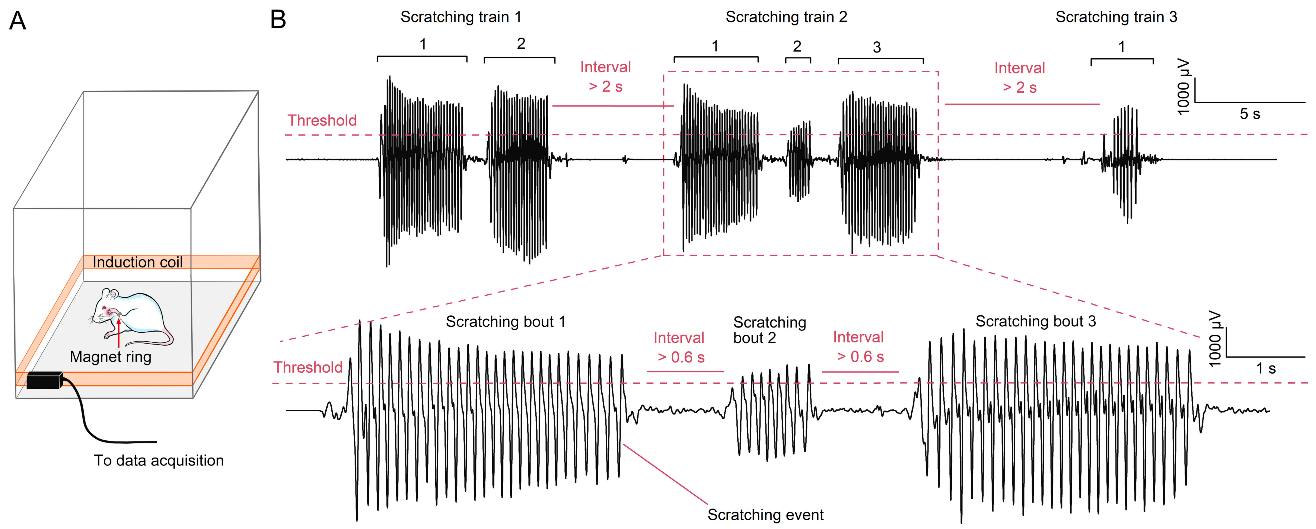


**Figure S1. Definition of itch-induced scratching behaviors.** **Related to Figure 1.**

(A) Schematic depicting the recording of scratching behaviors.

(B) An example trace of the rat hindpaw motion signal, illustrating the definition of scratching event, scratching bout and scratching train.

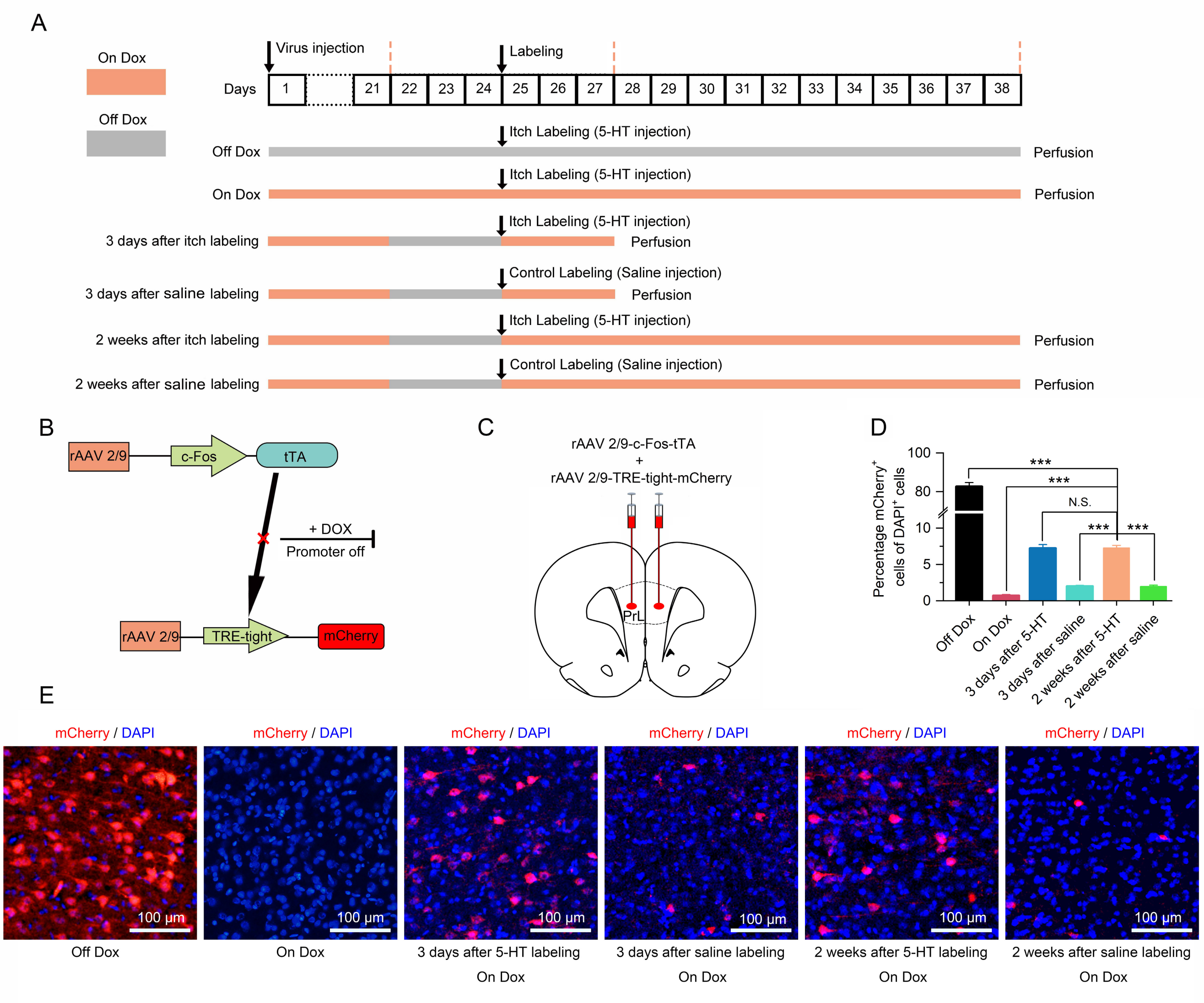


**Figure S2. Empirical proof of the validity and reliability of the Tet-Off system. Related to Figure 4.**

(A) Extended protocol and timeline of the experiments.

(B) Schematic showing the composition of the Tet-Off system.

(C) Schematic diagram showing bilateral injection of rAAV 2/9-c-Fos-tTA mixed with rAAV 2/9-TRE-tight-mCherry into the PrL.

(D) Percentage of neurons expressing mCherry under different manipulations. n = 4 rats in each group.

(E) Representative images of mCherry expression under various manipulations.

The data are presented as the mean ± SEM, and have a normal distribution. One-way ANOVA followed by the LSD post hoc test; N.S., not significant, ***$P < 0.001$.

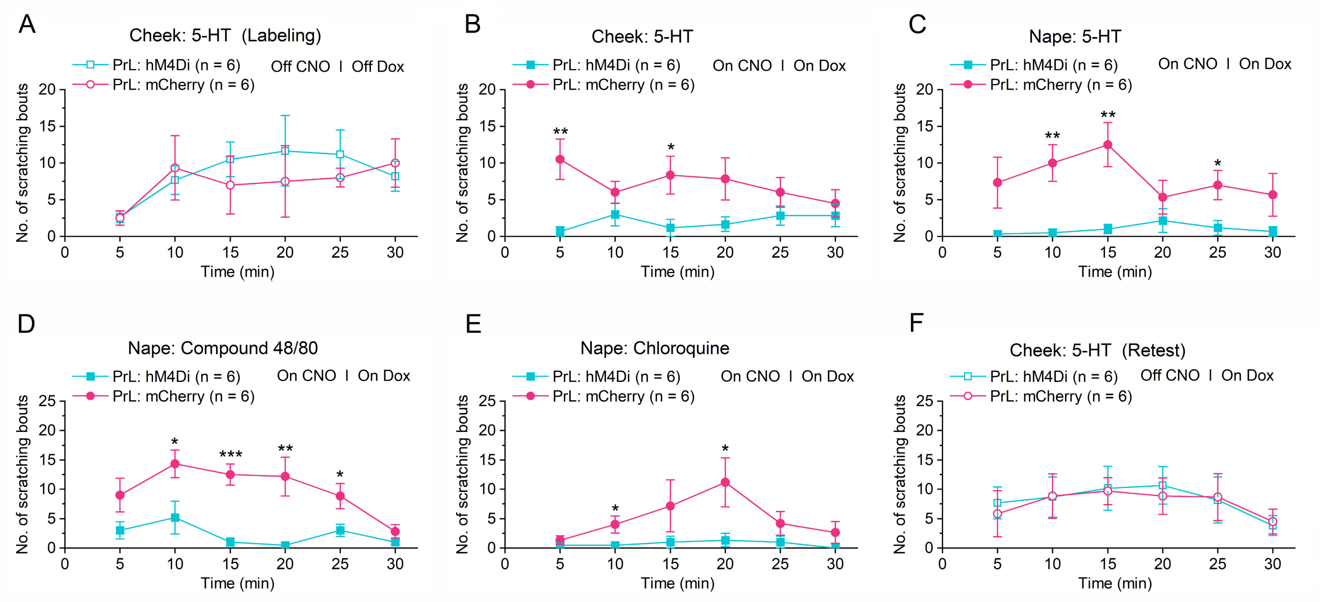


**Figure S3. Pharmacogenetic inhibition of itch-responsive neurons in the PrL decreased the number of scratching bouts induced by pruritogens. Related to Figure 4.**

(A) After Dox diet was stopped for 3 days, rats were injected with 5-HT into the cheek to label itch-responsive neurons. There was no significant difference in the number of scratching bouts between rats with hM4Di-mCherry$^{+}$ neurons and mCherry$^{+}$ neurons.

(B–E) In rats fed with a Dox diet, pharmacogenetic inhibition of itch-responsive neurons in the PrL significantly decreased the number of scratching bouts induced by intradermal injection of 5-HT (cheek, B; nape, C), compound 48/80 (nape, D), and chloroquine (nape, E).

(F) In rats fed with a Dox diet, there were no significant differences in the number of scratching bouts between the hM4Di and mCherry groups when CNO was not administered before the test.

The data are presented as the mean ± SEM, and have a normal distribution. Two-way repeated measures ANOVA followed by the separate one-way ANOVA; $*P < 0.05$, $**P < 0.01$, $***P < 0.001$.

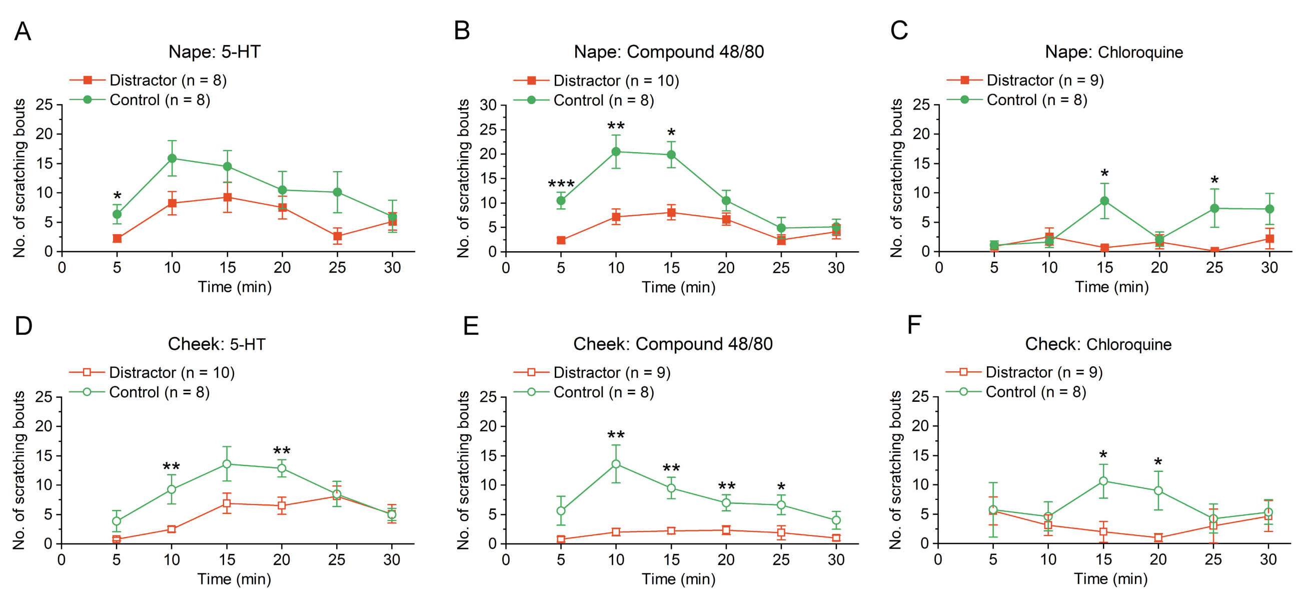


**Figure S4. A distracting stimulus decreased the number of scratching bouts induced by pruritogens. Related to Figures 5.**

(A–C) A distracting stimulus significantly decreased the number of scratching bouts induced by intradermal injection of 5-HT (A), compound 48/80 (B), or chloroquine (C) into the nape of the neck in rats.

(D–F) A distracting stimulus also significantly decreased the number of scratching bouts induced by intradermal injection of 5-HT (D), compound 48/80 (E), or chloroquine (F) in the rat cheek.

The data are presented as the mean ± SEM, and have a normal distribution. Two-way repeated measures ANOVA followed by the separate one-way ANOVA; *P < 0.05, **P < 0. 01, ***P < 0.001.

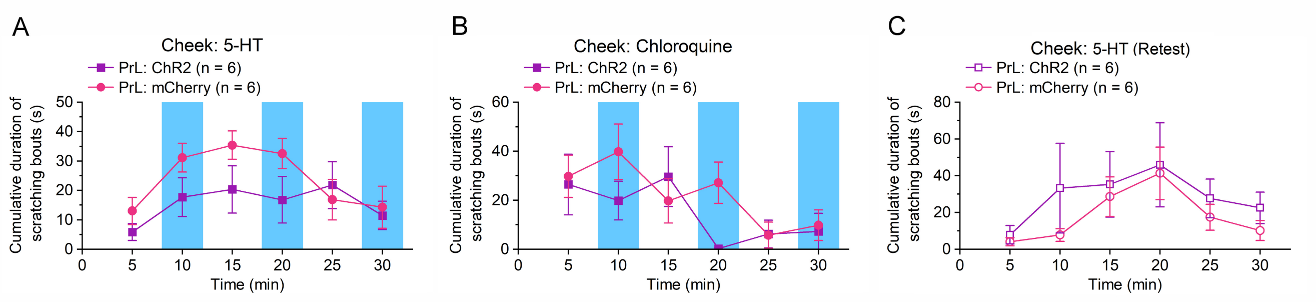


**Figure S5. The effects of optogenetic activation of attention-related neurons in the PrL on the number of scratching bouts induced by pruritogens. Related to Figure 6.**

(A, B) The effects of optogenetic activation of attention-related neurons on the number of scratching bouts induced by injection of 5-HT (cheek, A) and chloroquine (cheek, B).

(C) In rats fed with a Dox diet and not subjected to optogenetic activation, the number of scratching bouts in the ChR2 group was not significantly different from that in the control group.

The data are presented as the mean ± SEM.

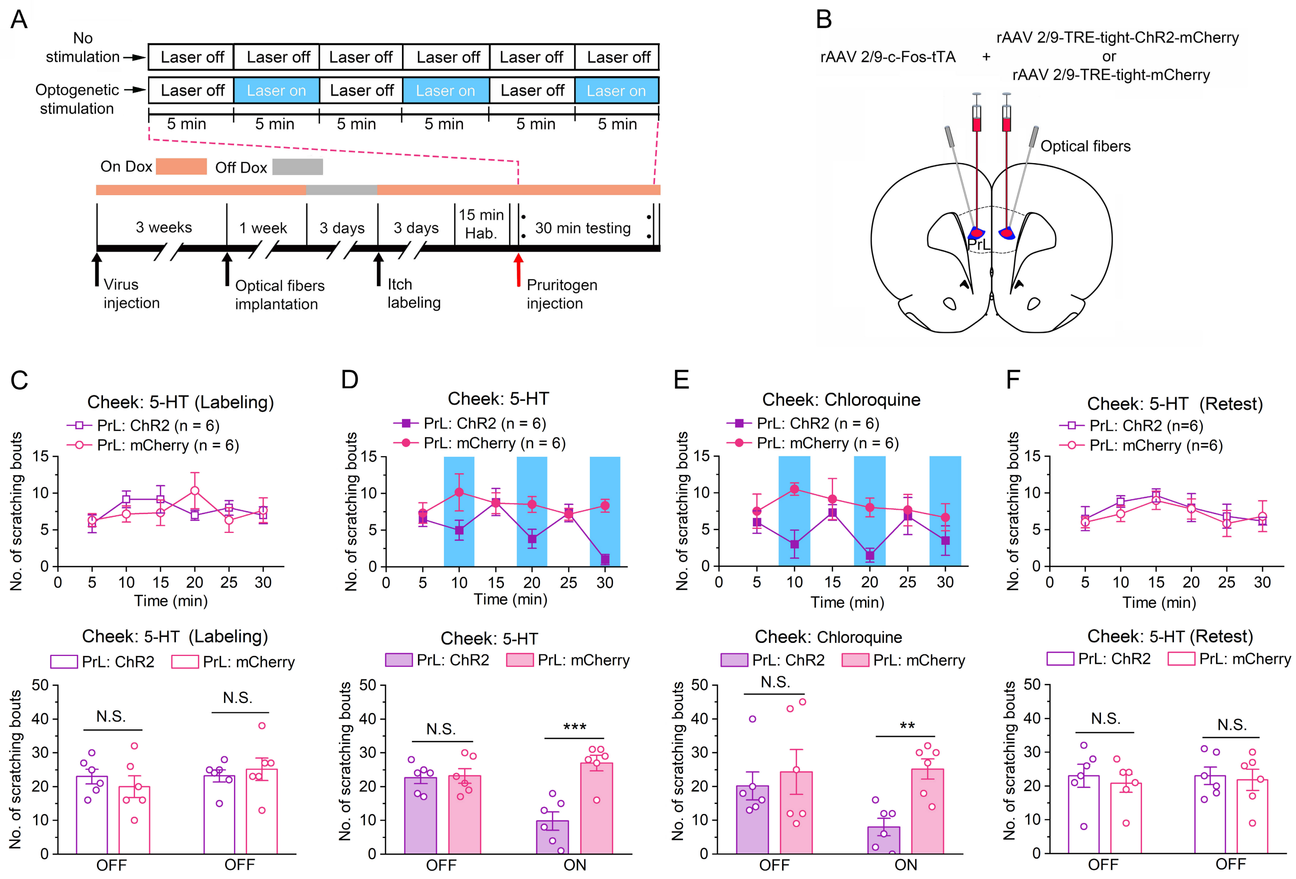


**Figure S6. Optogenetic stimulation of the itch-responsive neurons in the PrL suppressed itch processing. Related to Figure 4.**

(A) Flow diagram of the basic experimental timeline for itch-responsive neurons labeling and optogenetic activation manipulations.

(B) Schematic showing injection of rAAV 2/9-cFos-tTA mixed with rAAV 2/9-TRE-tight-ChR2-mCherry or pAAV 2/9-TRE-tight-mCherry and implantation of optical fibers in the bilateral PrL of rats.

(C) After 3 days without Dox diet, rats were injected with 5-HT into the cheek to label itch-responsive neurons in the PrL. n = 6 rats in each group.

(D, E) Optogenetic activation of itch-responsive neurons in the PrL significantly impaired scratching behaviors during acute itch induced by 5-HT (D) or chloroquine (E). n = 6 rats in each group.

(F) Rats fed with a Dox diet were injected with 5-HT in the absence of illumination to reassess the decrease in the number of scratching bouts resulting from optogenetic activation. n = 6 rats in each group.

The data are presented as the mean ± SEM, and have a normal distribution. Two-tailed Unpaired Student's t test; N.S., not significant, $^{**}P < 0.\ 01$, $^{***}P < 0.001$.

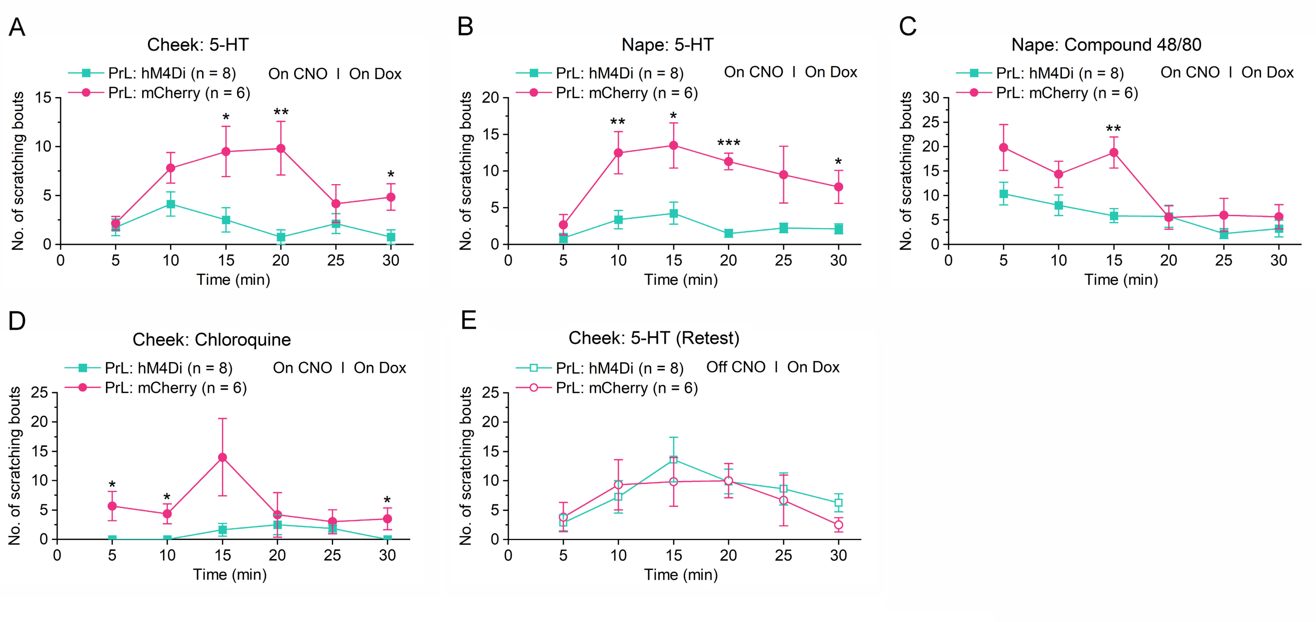


**Figure S7. Pharmacogenetic inhibition of attention-related neurons in the PrL decreased the number of scratching bouts induced by pruritogens. Related to Figure 6.**

(A–D) Dox diet was stopped for 3 days, a distracting stimulus was delivered to label attention-related neurons in the PrL. In rats fed with a Dox diet, pharmacogenetic inhibition of attention-related neurons significantly decreased the number of scratching bouts induced by intradermal injection of 5-HT (cheek, A; nape, B), compound 48/80 (nape, C), and chloroquine (nape, D).

(E) In rats fed with a Dox diet and not injected with CNO before the test, the number of scratching bouts in the hM4Di group was not significantly different from that in the control group.

The data are presented as the mean ± SEM, and have a normal distribution. Two-way repeated measures ANOVA followed by the separate one-way ANOVA; *$P < 0.05$, **$P < 0.\ 01$, ***$P < 0.001$.
